# Wdr5 and Myc cooperate to regulate formation of neural crest stem cells

**DOI:** 10.1242/dev.205204

**Published:** 2026-01-23

**Authors:** Karlin Compton, Elizabeth Barter, Carole LaBonne

**Affiliations:** ^1^ Department of Molecular Biosciences, Northwestern University, Evanston, IL 60208, USA; ^2^ National Institute for Theory and Mathematics in Biology, Northwestern University, Evanston, IL 60208, USA

**Keywords:** Wdr5, Myc, Pluripotency, Stem cell, *Xenopus*, Neural crest

## Abstract

Wdr5, a multifunctional scaffolding protein, with established roles in chromatin regulation and pluripotency, but its functions in early development remain poorly understood. Here, we show that *Xenopus wdr5* is expressed in blastula stem cells and enriched in neural crest cells. Depletion of *wdr5* abolished neural crest gene expression in embryos and in reprogrammed explants while expanding neural plate border and neural plate domains. Gain-of-function experiments revealed striking dose-dependent effects: low Wdr5 enhanced neural crest formation, whereas high levels suppressed it, suggesting a requirement for precise stoichiometry with interacting partners. We identify Myc as an essential co-factor for Wdr5 in neural crest – Wdr5 and Myc physically interact and co-expression at defined ratios rescues neural crest formation. We further show that the Wdr5 WBM site is required for Myc-dependent activation of neural crest genes, whereas the WIN site regulates *myc* expression itself; both domains are necessary to rescue *wdr5* depletion. These findings reveal that Wdr5 orchestrates neural crest development through multiple, domain-specific mechanisms, integrating stoichiometric control with partner-specific transcriptional regulation, and underscores the importance of precise co-factor ratios in cell fate decisions.

## Teaser

**Summary:** Wdr5 regulates neural crest development in *Xenopus* through dose-dependent, domain-specific interactions with Myc, integrating stoichiometric control and transcriptional regulation crucial for balanced cell fate specification.

## INTRODUCTION

The neural crest, a stem cell population unique to vertebrates, contributes a large and diverse set of cell types to the vertebrate body plan, including much of the peripheral nervous system, melanocytes, and craniofacial bone and cartilage (Le Douarin and Kalcheim, 1999). Because acquisition of these cells drove the evolution of vertebrates, a deeper understanding of the mechanisms regulating formation of neural crest cells will provide key insights into vertebrate origins, and how these cells contribute such a wide range of specialized cell types to the vertebrate body plan (Schock et al., 2023).

Neural crest cells arise in the ectoderm at the neural plate border (NPB) and retain multi-germ layer developmental potential beyond when most embryonic cells have become lineage restricted (Prasad et al., 2012). Insights into the origins of neural crest potential came from the realization that these stem cells share gene regulatory network components, and other features, with pluripotent stem cells of the vertebrate blastula (Buitrago-Delgado et al., 2015, 2018; York et al., 2024) Both pluripotent blastula and neural crest stem cells express core neural crest regulatory genes, such as *snai1*, *id3*, *foxd3*, *tfap2a*, as well as the core pluripotency factors *myc*, *ventx/nanog*, *pou5* and *sox2/3* (Bellmeyer et al., 2003; Buitrago-Delgado et al., 2015; York et al., 2024; Rigney et al., 2025). These two stem cell populations share additional features, such as high levels of Erk activity, low levels of histone acetylation and a requirement for BET family epigenetic readers (Geary and LaBonne, 2018; Huber et al., 2024; Rao and LaBonne, 2018). HDAC1 can enhance the generation of neural crest cells and has also been shown to cooperate with NANOG to promote pluripotency in murine and human embryonic stem cells (Bogdanovic et al., 2012; Dovey et al., 2010; Rao and LaBonne, 2018; Watanabe et al., 2013).

To gain further insights into the control of developmental potential in neural crest stem cells, we sought to identify new regulators of these stem cell populations. Using previously published datasets of the transcriptome changes that occur when blastula stem cell explants are reprogrammed to a neural crest state (Huber and LaBonne, 2024), we identified *wdr5* (*WD40 repeat domain containing protein 5*) as a factor significantly upregulated in response to neural crest induction. Wdr5 is a highly conserved protein that shares more than 90% sequence identity across vertebrates (Schuetz et al., 2006). It is known for scaffolding histone methyltransferase complexes (COMPASS) as well as for interacting with diverse transcription factors (Guarnaccia and Tansey, 2018). *Wdr5* is robustly expressed in mouse embryonic stem cells and its expression decreases as these cells differentiate (Ang et al., 2011). Wdr5 is involved in the formation of a number of histone-modifying complexes in stem cells, and may also play a role in reading histone modifications (Guarnaccia and Tansey, 2018; Ruthenburg et al., 2006; Schuetz et al., 2006; Wysocka et al., 2005).

Recent evidence indicates that the role of Wdr5 in depositing H3K4me3 explains only a subset of its effects on transcription, whereas others may be mediated through interactions with DNA-binding transcription factors (Siladi et al., 2022). Wdr5 has been shown to interact with a number of transcription factors, including p53, Twist, Pou5f1/Oct4 and Myc (Ang et al., 2011). Physical interactions with Wdr5 are largely mediated by one of two highly conserved binding sites on opposite sides of the β-propeller structure of Wdr5: the Wdr5 binding motif (WBM) or the Wdr5 interaction domain (WIN) (Dharmarajan et al., 2012; Guarnaccia and Tansey, 2018; Thomas et al., 2015b).

In this study, we investigate the role of Wdr5 in neural crest stem cells in *Xenopus*. We show that *wdr5* is required for formation of the neural crest in whole embryos and that depletion of *wdr5* prevents pluripotent blastula explants from adopting a neural crest fate. Additionally, we show that exogenous *wdr5* expression alters neural crest factor expression in a dose-dependent manner, with higher concentrations exhibiting an inhibitory effect on neural crest gene expression, suggesting stoichiometric effects. We show that Wdr5 and the pluripotency/neural crest transcription factor Myc physically interact with one another in *Xenopus* and colocalize to the same cells at neural crest stages. Co-expression of *wdr5* and *myc* together facilitates an expansion of the neural crest domain. Finally, we show find that mutations in either of the two conserved interaction domains alters the ability of Wdr5 to regulate neural crest formation in distinct ways, and that binding between Wdr5 and Myc is required for the regulation of neural crest formation.

## RESULTS

### *wdr5* is expressed in blastula stem cells and is upregulated in neural crest cells

**Fig. 1.**
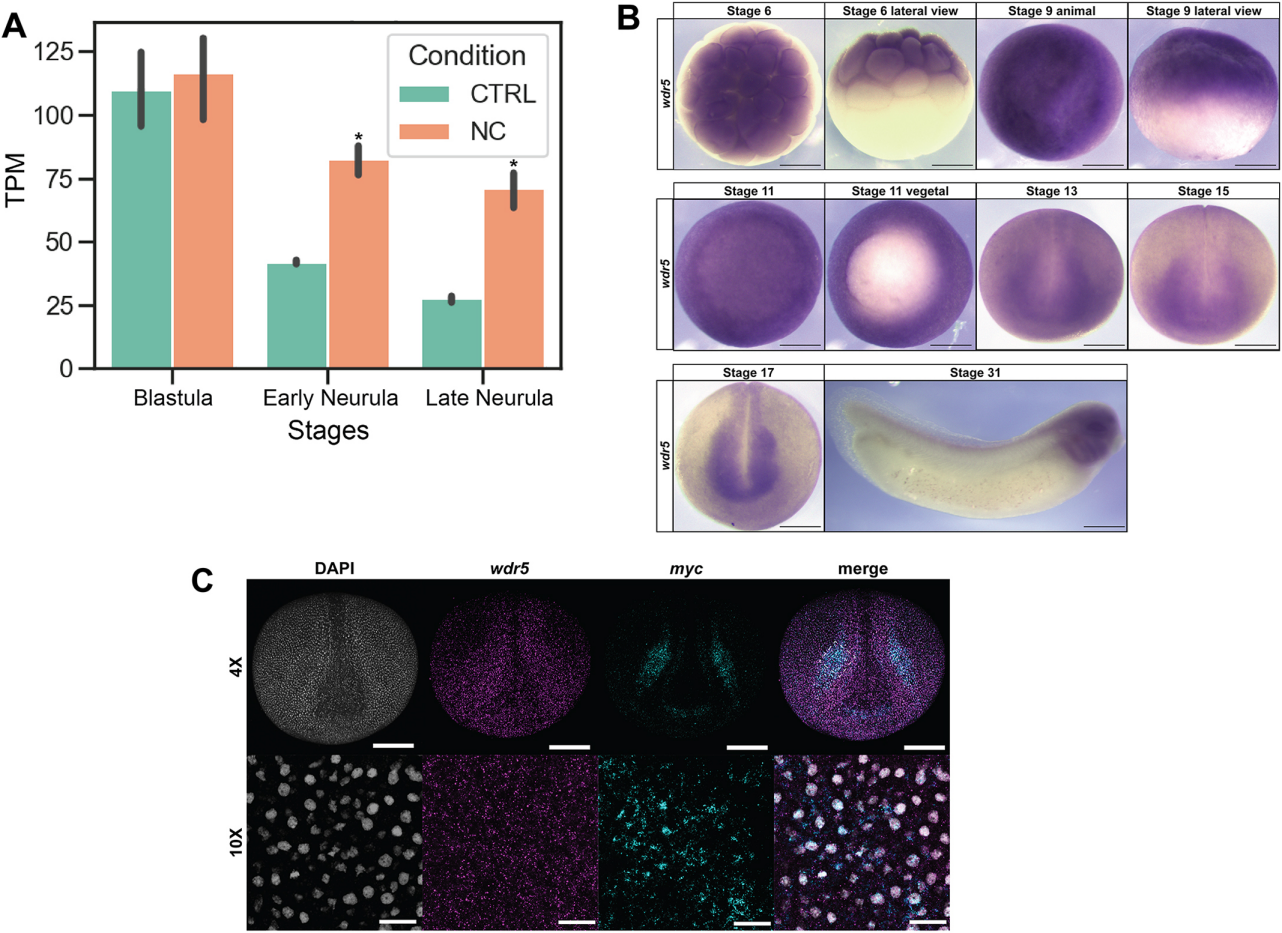
**Characterization of *wdr5* expression.** (A) Analysis of previously published RNA-Seq data (York et al., 2024) shows *wdr5* transcripts are abundant in the animal pole cells of the blastula stages and differentially enriched in neural crest-induced animal caps at early and late neurula stages (*P<0.05). (B) Spatial and temporal expression of *wdr5* shows that *wdr5* transcripts are maternally provided, enriched in blastula stem cells and retained broadly throughout the neuroectoderm into developing neural crest stem cells and neural crest derivatives. (C) Fluorescent *in situ* hybridization chain reaction confirms *wdr5* expression in the neural crest that colocalizes with *myc* – a canonical neural crest and pluripotency factor. CTRL, control; NC, neural crest; TPM, transcripts per million. Scale bars: 250 μm in B and C (10× images); 100 μm in C (4× images).

As a first step in studying the role of *wdr5* in early embryonic *Xenopus* development we characterized its expression. Analysis of previously published RNA sequencing (RNA-Seq) data (Huber and LaBonne, 2024; Johnson et al., 2022) showed that *wdr5* is highly expressed in the animal pole cells of cleavage stage embryos and blastula stems cells, at levels comparable to factors involved in pluripotency, such as *ventx2.2* and *tfap2a*. Wnt/Chordin mediated-reprogramming of animal pole explants to a neural crest state promoted expression of *wdr5* at both early and late neurula stages (Fig. 1A). We used whole-mount *in situ* hybridization (WISH) to examine the spatial expression of *wdr5*. Consistent with the RNA-Seq data, *wdr5* was strongly expressed in animal pole cells of cleavage-stage embryos and in the pluripotent animal pole cells of blastula-stage embryos (Fig. 1B). At early and mid-neurula stages, *wdr5* expression is enriched in the developing CNS and neural crest cells but is also expressed broadly throughout the ectoderm. At tailbud stages, *wdr5* is enriched in migratory neural crest cells, and is also expressed in the developing ear as previously reported (Bibonne et al., 2013). We used fluorescent *in situ* hybridization chain reaction to examine whether *wdr5* is co-expressed with neural crest factors. We observed that *wdr5* is co-expressed with *myc* in the neural crest at neurula stages (Fig. 1C). Taken together, the spatial and temporal expression profile of *wdr5* is consistent with a role in regulation of neural crest development.

### *wdr5* is essential for neural crest formation and patterning of the NPB

**Fig. 2.**
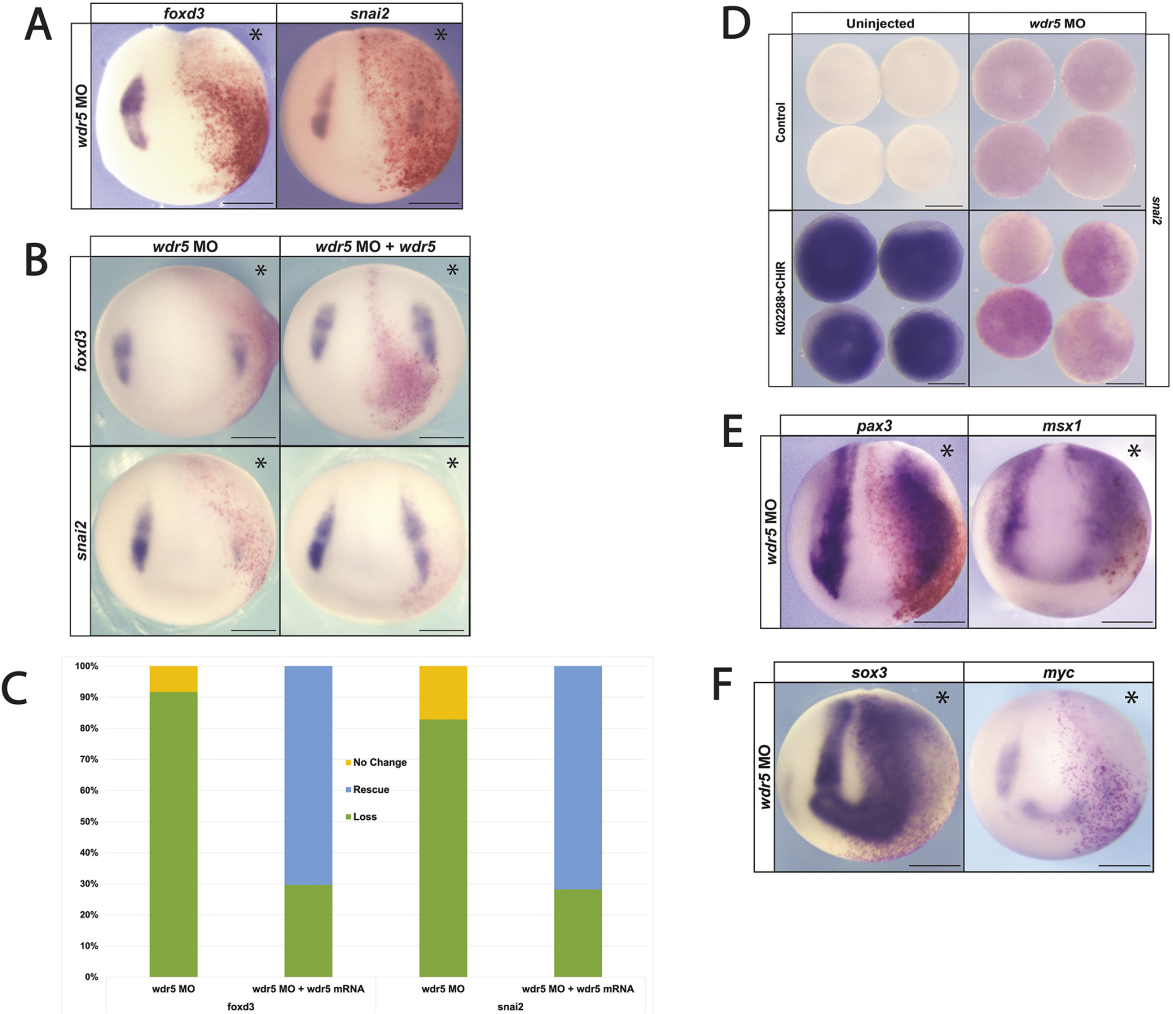
**Morpholino-mediated knockdown shows that *wdr5* is required for NC gene expression.** (A) Morpholino-mediated depletion of *wdr5* inhibits neural crest factor expression. (B) Expression of *wdr5* mRNA rescues morpholino-mediated neural crest gene expression. (C) Quantification of rescue percentage for the experiment shown in B. (D) Morpholino-mediated depletion of *wdr5* prevents pluripotent blastula cells from being reprogrammed to neural crest state. (E) Morpholino-mediated depletion of *wdr5* does not inhibit expression of NPB and neural plate factors. (F) Knockdown of *wdr5* leads to an expansion of the neural plate factor *sox3* and loss of *myc* expression at neurula stages. Asterisks indicate the injected side of the embryo. Scale bars: 250 μm.

After finding that *wdr5* expression is enriched in neural crest cells, we next examined whether it is essential for neural crest formation. To test this, we designed a *wdr5* translation-blocking morpholino oligonucleotide (MO) that targets both allo-alleles of *wdr5*. Co-injection of the MO with mRNA encoding a C-terminally Myc-tagged Wdr5 led to loss of Wdr5 expression as assayed by western blot (Fig. S1D). We performed targeted injection into two animal pole blastomeres on one side of 8-cell *Xenopus* embryos to target the neural crest, with β-galactosidase co-injected as a lineage tracer. Embryos were cultured to neurula stages and examined by WISH for expression of neural crest markers. Depletion of *wdr5* resulted in a loss of expression of the neural crest factors *foxd3* (86%, *n*=74) and *snai2* (94%, *n*=44), indicating that Wdr5 activity is essential for neural crest formation (Fig. 2A; Fig. S1A). Neural crest could be rescued by co-injecting mRNA encoding N-terminally Flag-tagged Wdr5 (*foxd3* 70% rescued, *n*=54; *snai2*: 72% rescued, *n*=39) compared to the loss of neural crest factor expression in embryos injected with only *wdr5* MO (*foxd3*: 92% loss, *n*=60; *snai2*: 82% loss, *n*=35) (Fig. 2B,C; Fig. S1C).

To determine whether *wdr5* is also required for reprogramming pluripotent blastula cells to a neural crest state, we treated explants from control or morpholino-injected embryos with a small molecule inhibitor of BMP signaling (K02288) and an agonist of Wnt signaling (CHIR99021) to induce neural crest (Huber and LaBonne, 2024). Morpholino-mediated depletion of *wdr5* prevented reprogramming to a neural crest state, as evidenced by a failure to induce the expression of *snai2* (92.5%, *n*=54) compared to controls (Fig. 2D; Fig. S1B).

### Loss of *wdr5* enhances NPB formation

We next investigated whether *wdr5* is specifically required for neural crest gene expression or alternatively is also essential for establishing the NPB region. The NPB is characterized by the overlapping expression of several transcription factors, including *pax3*, *zic1* and *msx1* (Groves and LaBonne, 2014). Surprisingly, we found that depletion of *wdr5* led to expanded expression of the NPB factors *pax3* (85% expansion, *n*=47) and *msx1* (82% expansion, *n*=46), as well as neural plate marker *sox3* (93% expansion, *n*=42), into regions that normally form neural crest and epidermis (Fig. 2E,F; Fig. S1A). Thus, *wdr5* is not required for formation of all ectoderm-derived cell types. Interestingly, expression of *myc* at the NPB did require *wdr5* (90% loss, *n*=54).

### Morpholino-mediated *wdr5* depletion does not significantly impact global H3K4me3 levels

Wdr5 can serve as a scaffolding protein for MLL/SET methyltransferases, which facilitate trimethylation of histone 3 at lysine 4 (H3K4me3), an epigenetic modification associated with transcriptional activation (Bernstein et al., 2002; Dou et al., 2006). We therefore examined the effects of *wdr5* depletion on global methylation levels by comparing H3K4me3 levels in control explants with explants depleted for *wdr5*. Control embryos or embryos injected with *wdr5* morpholino in both cells at the 2-cell stage were cultured to blastula stages when animal caps were excised and cultured to neurula stages. Western blot analysis showed that *wdr5*-depleted explants and control explants exhibited comparable levels of H3K4me3 as normalized to total histone H3 (Fig. S1F,G). Thus, the loss of neural crest cells in *wdr5*-depleted embryos is independent of major changes in global methylation levels.

### Wdr5 activity affects neural crest formation in a dose-dependent manner

**Fig. 3.**
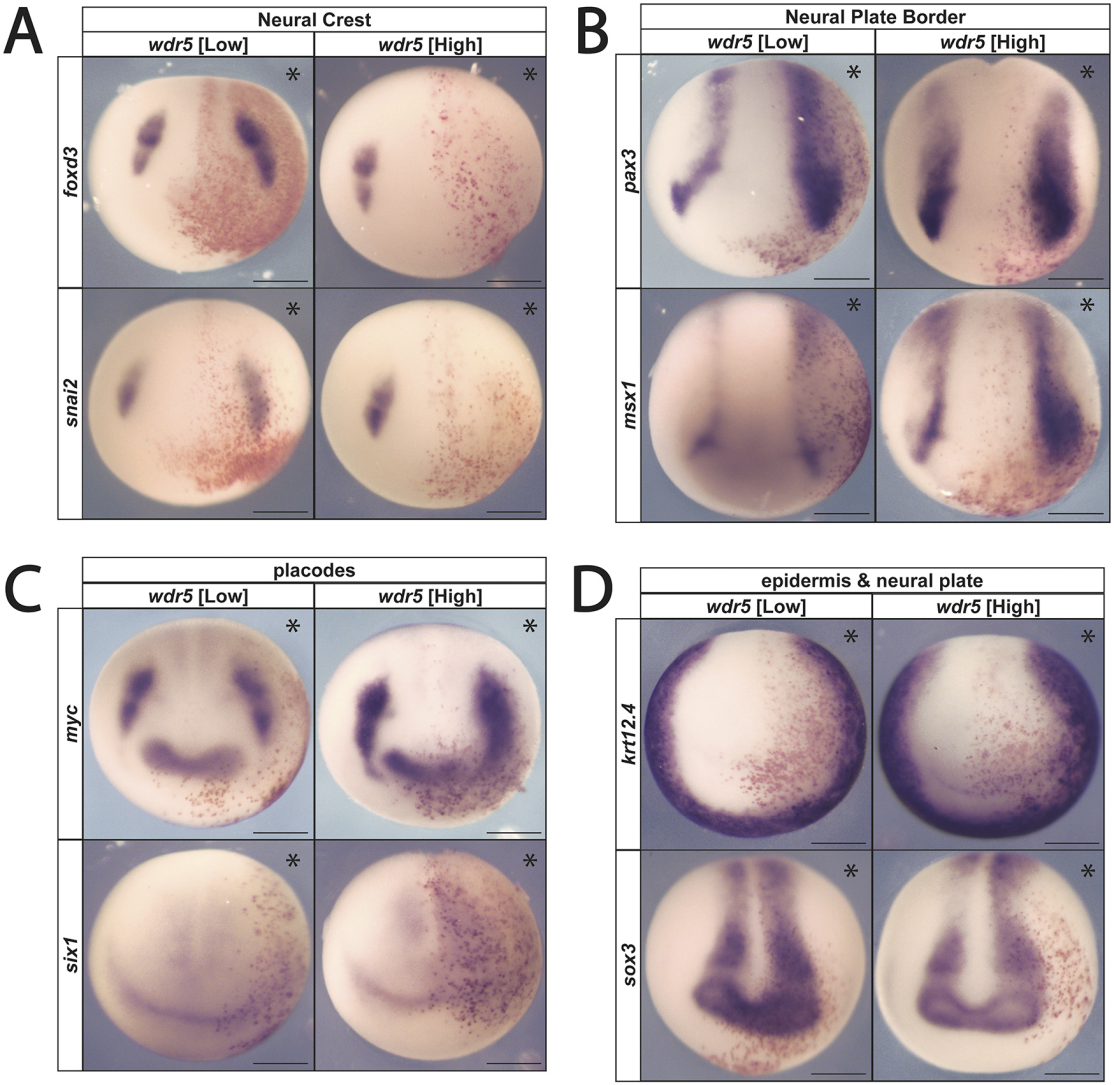
**Overexpression of *wdr5* exhibits concentration-dependent effects on neural crest factor expression.** (A) Exogenous *wdr5* mRNA shows concentration-dependent effects on neural crest gene expression. (B) Increased *wdr5* expression expands NPB gene expression independently of mRNA concentration. (C) Exogenous *wdr5* mRNA exhibits expanded expression of *myc* expression and inhibition of *six1* expression. (D) Increased *wdr5* expression inhibits expression of epidermal keratin factor (*krt12.4*) and expansion of the neural plate factor *sox3* independently of mRNA concentration. Asterisks indicate the injected side of the embryo. Scale bars: 250 μm.

Given that *wdr5* is required for neural crest formation, we next examined whether increasing *wdr5* levels might promote neural crest formation. mRNA encoding Wdr5 was injected unilaterally into two animal pole cells of 4-cell *Xenopus* embryos targeting the presumptive neural crest, with the uninjected side serving as an internal control. Injections were carried out using a range of mRNA concentrations to identify potential dose-dependent effects. Lower levels of *wdr5* were found to enhance the expression of the neural crest factors *foxd3* (71%, *n*=54) and *snai2* (78%, *n*=48) and the neural plate border markers *pax3* (90%, *n*=83) and *msx1* (87%, *n*=62) (Fig. 3A,B). However, whereas higher levels of *wdr5* similarly enhanced neural plate border gene expression (*pax3*: 934% expansion, *n*=48; *msx1*: 90% expansion, *n*=40) those same doses inhibited expression of neural crest markers (*foxd3*, 78%, *n*=49; *snai2*, 89%, *n*=51) (Fig. 3A,B; Fig. S2). The striking concentration-dependent effects of *wdr5* on neural crest factors suggests that its function is likely dependent on interaction partners sensitive to stoichiometry. Both concentrations inhibited expression of placodal markers (Fig. 3C). By contrast, the observed enhancement of neural plate border gene expression by both high and low levels of *wdr5* indicates that *wdr5* functions somewhat differently in the regulation of these two cell types.

### Wdr5 and Myc physically interact

**Fig. 4.**
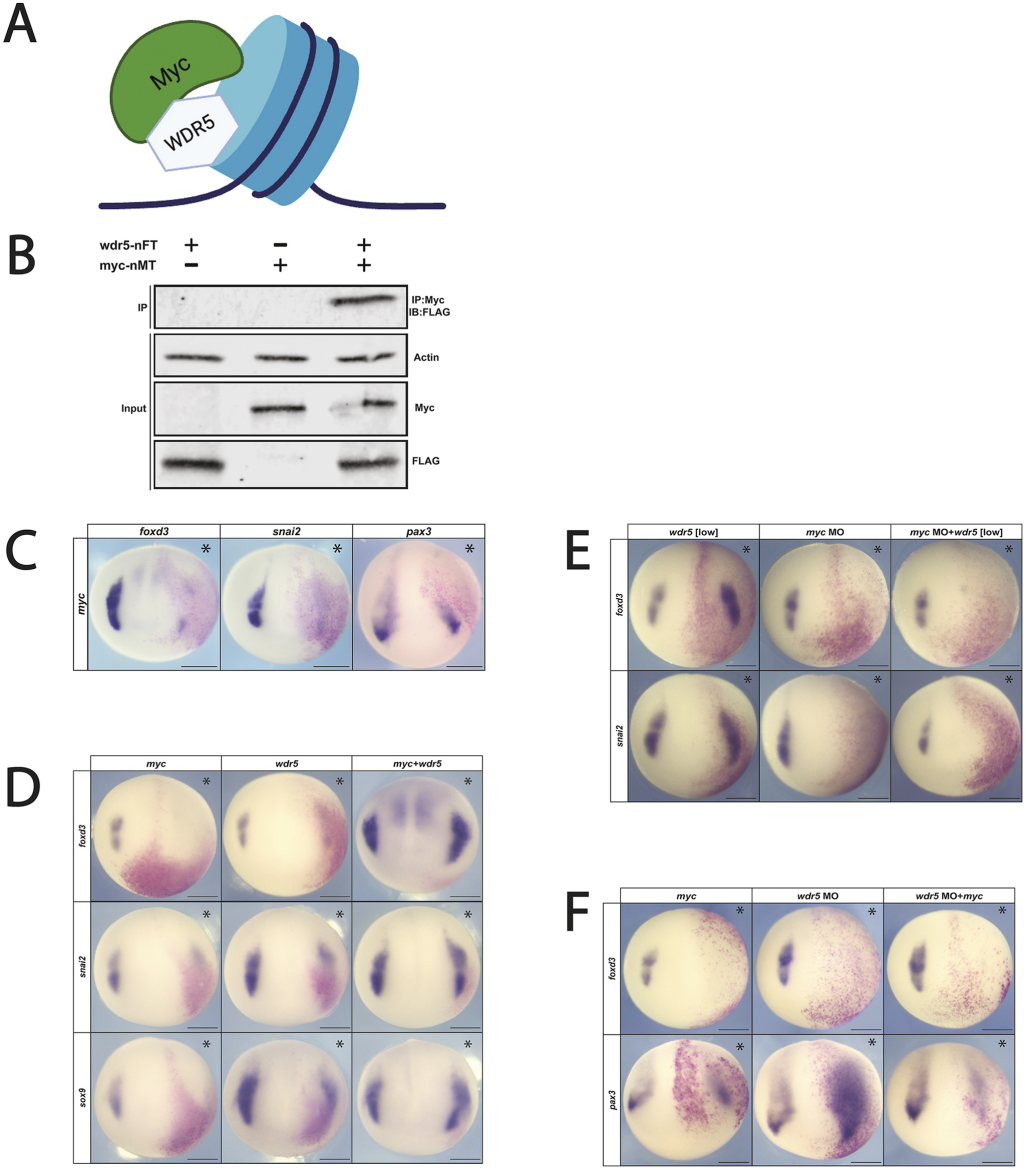
**Myc and Wdr5 interact directly to facilitate neural crest factor expression.** (A) Wdr5 and Myc directly interact at chromatin to influence downstream gene expression of Myc target genes. (B) Western blot of Co-IP shows that FLAG-tagged Wdr5 (wdr5-nFT) and Myc-tagged Myc (myc-nMT) directly interact in *Xenopus* embryos. (C) *myc* mRNA overexpression inhibits neural crest and NPB gene expression. (D) Co- expression of *wdr5* mRNA and *myc* mRNA facilitates neural crest gene expression. (E) *myc* expression is required for Wdr5-mediated neural crest expansion. (F) *myc* mRNA inhibits neural crest and NPB gene expression when *wdr5* expression is downregulated. Asterisks indicate the injected side of the embryo. Scale bars: 250 μm.

We have previously shown that the proto-oncogene Myc is required for neural crest formation (Bellmeyer et al., 2003; Light et al., 2005). Myc is also a known Wdr5-interacting factor that binds to its WBM site (Fig. 4A). WDR5 has been shown to interact directly with MYC in human cancer stem cells, and is required for MYC-mediated transcriptional regulation (Thomas et al., 2025a,b, 2019). Accordingly, we examined whether Wdr5 and Myc can physically interact in *Xenopus*. mRNA encoding Flag-tagged Wdr5 and Myc-tagged Myc was injected into one cell of 2-cell *Xenopus* embryos, embryos were lysed at blastula stages, and Myc was immunoprecipitated from lysates using an anti-Myc antibody. Western blot analysis using an anti-Flag antibody showed a robust physical interaction between Wdr5 and Myc (Fig. 4B).

### Myc overexpression inhibits neural crest formation

As a first step toward examining whether there is a joint role for Wdr5 and Myc in neural crest formation, we examined the effects of Myc upregulation on neural crest and NPB formation. mRNA encoding Myc was injected at a range of concentrations into two blastomeres of 4-cell *Xenopus* embryos targeting the presumptive neural crest. We found that at all concentrations of Myc inhibited expression of the neural crest markers *foxd3* (84% loss, *n*=48) and *snai2* (78% loss, *n*=45) (Fig. 4C). Surprisingly, Myc upregulation also reduced expression of the NPB factor *pax3* (Fig. 4C; Fig. S3A) (91% loss, *n*=29), in contrast to what was observed for Wdr5 gain of function. This finding is consistent with Myc having both Wdr5-dependent and -independent functions.

### Co-expression of Wdr5 and Myc promotes neural crest formation

If the relative expression levels of Myc and Wdr5 are important for their role in promoting neural crest formation, which could explain why increasing the levels of only one of them inhibits neural crest gene expression. We therefore investigated what the consequences of increased expression of both factors would be for neural crest formation. Embryos were injected in two cells at the 4-cell stage with either *myc* mRNA alone, *wdr5* mRNA alone (each at concentrations that decrease neural crest gene expression), or both *myc* and *wdr5* mRNA together at those concentrations. Strikingly, when injected together at concentrations at which they each inhibit neural crest formation, they instead expanded the neural crest domain, as evidenced by increased expression of *foxd3* (75%, *n*=42) and *snai2* (74%, *n*=50), as well as the SoxE transcription factor *sox9* (83%, *n*=33) (Fig. 4D). Together, these findings support the hypothesis that Wdr5 and Myc work together to promote neural crest formation.

To further test this hypothesis, we examined whether Myc was required for the ability of low levels of Wdr5 to enhance neural crest formation. Embryos were injected with *myc* MO, low levels of *wdr5* mRNA or both. Depletion of *myc* blocked neural crest formation even in embryos that expressed *wdr5* at neural crest-promoting levels (*foxd3*: *myc* MO 82%, *n*=44; *myc* MO+*wdr5*: 77%, *n*=43; *snai2*: *myc* MO 71%, *n*=15; *myc* MO+*wdr5*: 77%, *n*=22) (Fig. 4E; Fig. S3C). These results further demonstrate that Wdr5 and Myc function together to positively regulate neural crest formation.

As *wdr5* depletion expands the NPB (Fig. 2E), this suggests that endogenous Wdr5 functions to restrict the size of the NPB. We therefore examined what effect increasing *myc* levels would have on this expansion. Embryos were injected with *wdr5* MO, mRNA encoding *myc* or both *myc* and *wdr5* and cultured to neurula stages. Increased *myc* expression inhibited expression of *pax3* in both the presence or absence of *wdr5* depletion (*myc* only: 66.7%, *n*=45; *myc*+*wdr5* MO: 85.1%, *n*=47) (Fig. 4F; Fig. S3D). This suggests that the relative expression levels of *myc* and *wdr5* are crucial for proper patterning of the ectoderm.

### Mutations in Wdr5 binding sites reduce binding affinity to known Wdr5 binding partners

Wdr5 binds to its partners through one of two conserved binding sites, the WBM or the WIN (Guarnaccia and Tansey, 2018), which reside on opposite ends of the Wdr5 β-propellor structure. The WBM site has been implicated in binding to sequence-specific transcription factors, such as Myc and Oct4 (Pou5f3) (Ang et al., 2011; Thomas et al., 2019) whereas the WIN site binds to arginine containing motifs (consensus ‘ARA’) present in binding partners such as Kansl1 (Dias et al., 2014) and ‘canonical’ MLL/SET domain methyltransferases, and also mediates binding to histone H3 and chromatin (Dharmarajan et al., 2012; Patel et al., 2008a,b; Zhang et al., 2012).

**Fig. 5.**
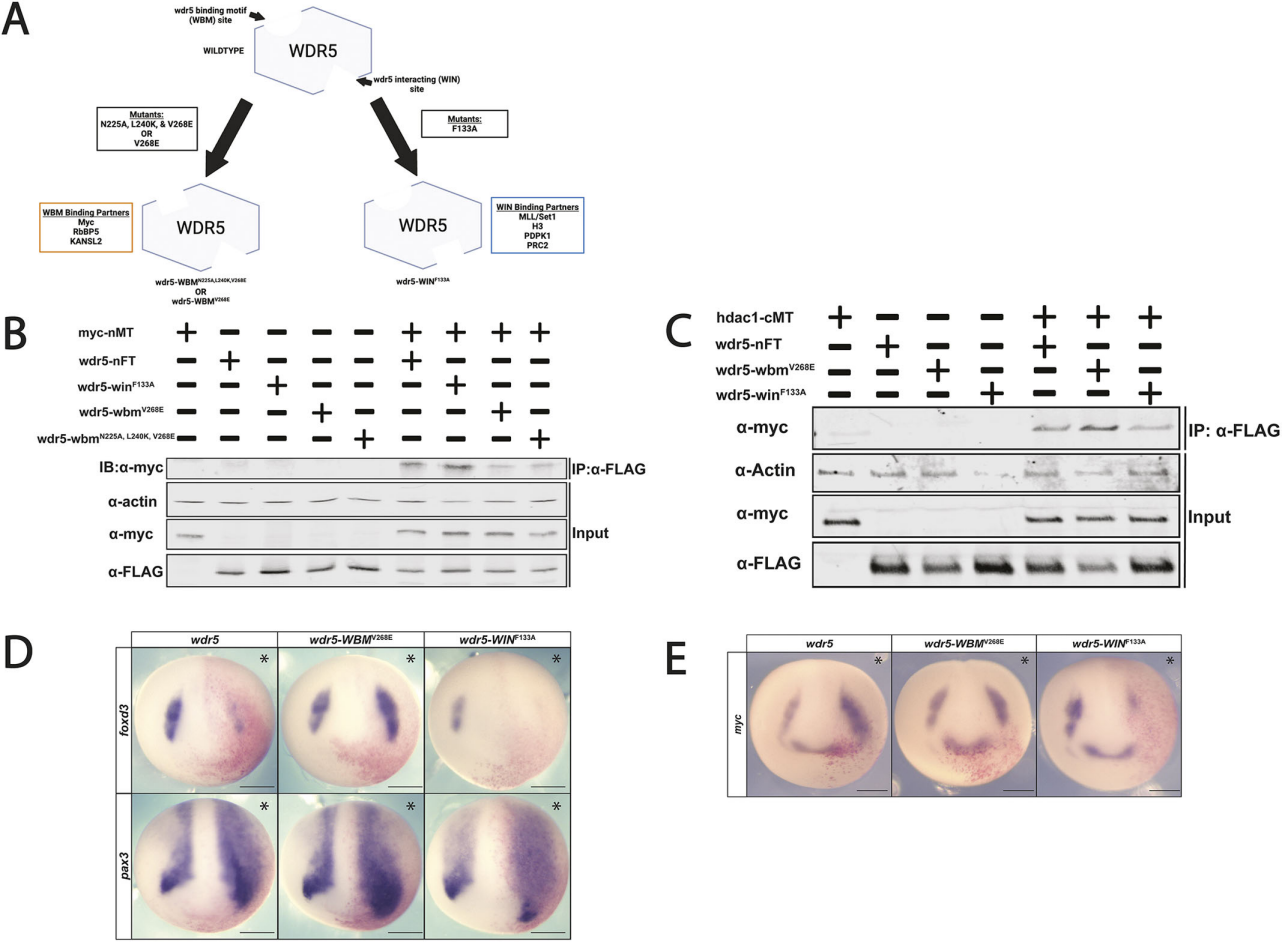
**Mutations in conserved binding sites on Wdr5 differentially affect neural crest gene expression.** (A) Wdr5 function is primarily mediated through two conserved and distinct binding sites, the Wdr5 binding motif (WBM) and the Wdr5 interacting domain (WIN), both of which can be disrupted by point mutations. (B) Western blot showcasing disruption of binding of Myc to Wdr5-WBM^V268^ and Wdr5-WBM^N225A,L240K,V268E^ in *Xenopus* embryos. (C) Western blot showcasing disruption of binding of Hdac1 to Wdr5-WIN^F133A^ in *Xenopus* embryos. (D) *wdr5-WBM^V268^* leads to an expansion of neural crest gene expression when expressed at levels that lead to loss of neural crest gene expression in *wdr5* and *wdr5-WIN^F133A^* embryos. Overexpression of *wdr5* and all resultant mutants expand NPB factor expression. (E) *wdr5* mutant overexpression differentially affects *myc* expression compared to wild-type *wdr5.* Asterisks indicate the injected side of the embryo. Scale bars: 250 μm.

Point mutations have been identified that can disrupt the interaction between Wdr5 and Myc in cancer cells (Thomas et al., 2019) (Fig. 5A). To determine whether these mutations can also disrupt interactions between these factors in early embryos, we used site-directed mutagenesis to substitute key residues in the highly conserved WBM and WIN sites. These mutants or wild-type Wdr5 were then co-expressed with Myc in early embryos, and co-immunoprecipitation (Co-IP) assays were carried out on blastula-stage lysates. Two different WBM mutants with one or three substitutions, respectively, WBM^V268E^ and WBM^N225A, L240K, V268E^, were found to reduce the interaction with Myc, whereas WIN^F133A^ did not (Fig. 5B,C; Fig. S4C,D). By contrast, WIN^F133A^ decreased interaction with Hdac1 while mutation of the WBM site increased this interaction (Fig. S4E,F).

### Wdr5 binding mutants differentially affect regulation of the neural crest

Given that mutation of the WBM binding site decreased interaction between Wdr5 and Myc, and that mutation of the WIN binding site decreased interaction between Wdr5 and Hdac1, we next compared the ability of these mutants to regulate NPB and neural crest formation as compared to wild-type Wdr5. Wdr5-WBM^V268E^, Wdr5-WIN^F133A^ or Wdr5 were expressed unilaterally at equivalent levels (Fig. S4B) and injected embryos were cultured to neural plate stages for WISH. Strikingly, whereas Wdr5-WIN^F133A^, like wild-type wdr5, led to a loss of neural crest gene expression (Wdr5-WIN^F133A^ *foxd3*: 84.6% loss, *n*=52), Wdr5-WBM^V268E^ enhanced neural crest formation (Wdr5-WBM^V268E^ *foxd3*: 66% expansion, *n*=50) (Fig. 5D). This is likely because its reduced ability to interact with Myc leads to Wdr5-WBM^V268E^ functioning much like lower levels of Wdr5. By contrast, expression of the NPB factor *pax3* was expanded by all three Wdr5 isoforms (*pax3*: Wdr5 92.6% expansion, *n*=54; Wdr5-WIN^F133A^ 91% expansion, *n*=45; Wdr5-WBM^V268E^ 88%, *n*=74); this provides further evidence that the mechanisms through which Wdr5 enhances NPB formation are distinct from those regulating neural crest formation.

Given that Wdr5 increased the neural crest domain of Myc at neural plate stages, we next examined what effects the WBM and WIN domain mutants would have. Strikingly, expression of Wdr5-WIN^F133A^ inhibited *myc* expression (*myc*: 81% loss, *n*=66) unlike wild-type Wdr5 (*myc*: 84.6%, *n*=39) or Wdr5-WBM^V268E^ (*myc*: 77.2%, *n*=66), which increased *myc* expression (Fig. 5E; Fig. S4A).

Together, these findings indicate that the WBM and WIN domains each mediate a subset of Wdr5 activities, with the WBM domain required for regulating definitive neural crest genes in partnership with Myc. By contrast, Myc itself is regulated by WIN-mediated functions of Wdr5 that are independent of WBM–Myc binding, and this regulation appears to be less dependent upon stoichiometry.

### Both the WBM and WIN sites are required to rescue the effects of *wdr5* depletion

**Fig. 6.**
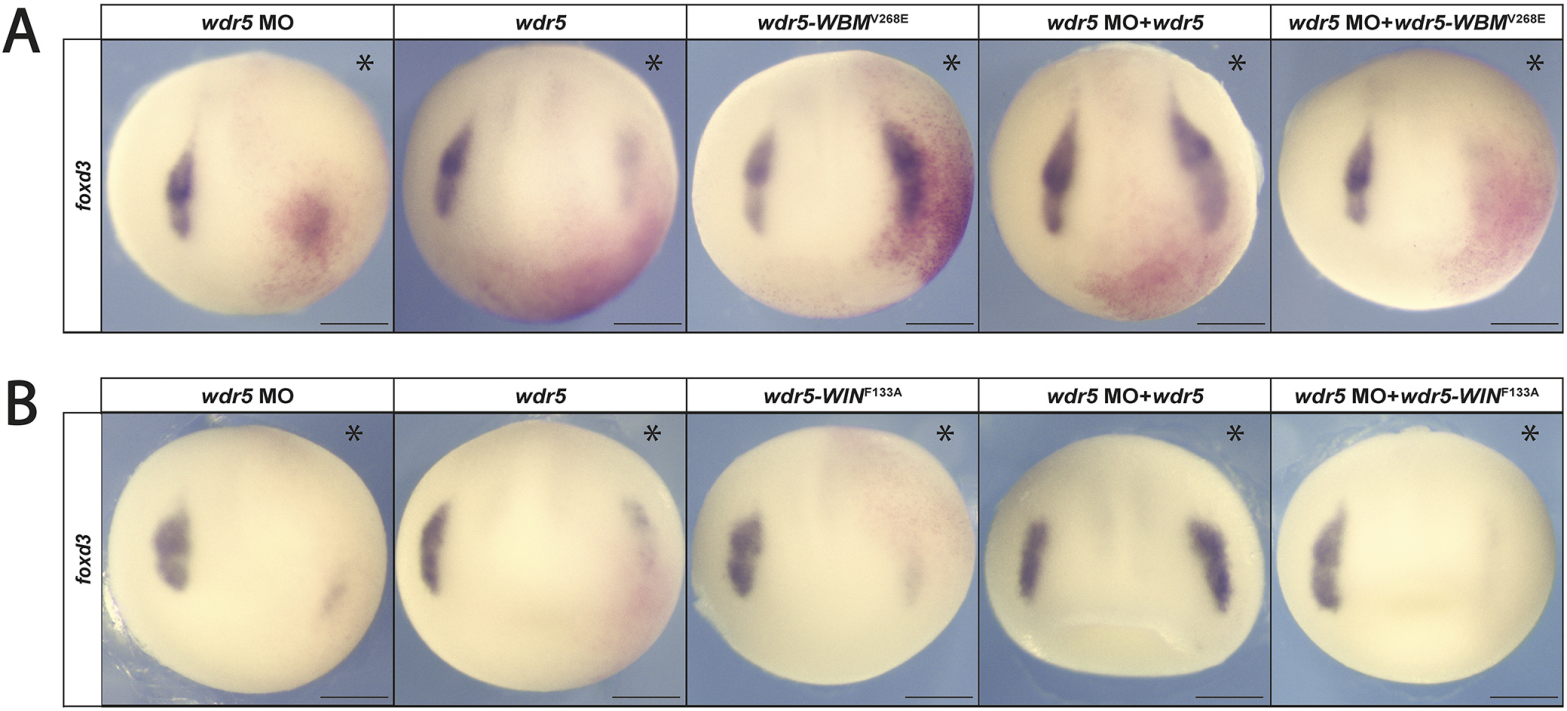
**Both Wdr5 binding sites are required for the expression of neural crest genes.** (A) *wdr5-WBM^V268E^* overexpression fails to rescue neural crest expression in the absence of endogenous *wdr5* expression. (B) *wdr5-WIN^F133A^* overexpression fails to rescue neural crest expression in the absence of endogenous *wdr5* expression. Asterisks indicate the injected side of the embryo. Scale bars: 250 μm.

Given the difference in phenotypes observed for the Wdr5 binding domain mutants, we wished to determine the extent to which either the WBM or WIN mutant could rescue the effects of *wdr5* depletion. To test this, we co-injected embryos depleted for *wdr5* with mRNA encoding Wdr5 at a concentration that on its own inhibits neural crest formation, or with the WBM or WIN mutants expressed at equivalent levels (Fig. S5C,D). We observed that, although the WBM mutant on its own expands neural crest, it was unable to fully rescue neural crest formation in *wdr5*-depleted embryos as evidenced by lack of rescue of *foxd3* expression (*wdr5* MO+*wdr5-WBM^V268E^* mRNA: 87% failed to rescue, *n*=37) (Fig. 6A; Fig. S5A). Similarly, the WIN domain mutant was also unable to rescue wild-type *wdr5* depletion (*wdr5* MO+*wdr5-WIN^F133A^* mRNA: 89% failed to rescue, *n*=53) (Fig. 6B; Fig. S5B). Taken together, the insufficiency of either mutant to rescue the loss-of-function phenotype indicates that both conserved binding sites of Wdr5 are required for neural crest formation.

### Lowering Myc levels promotes neural crest formation

**Fig. 7.**
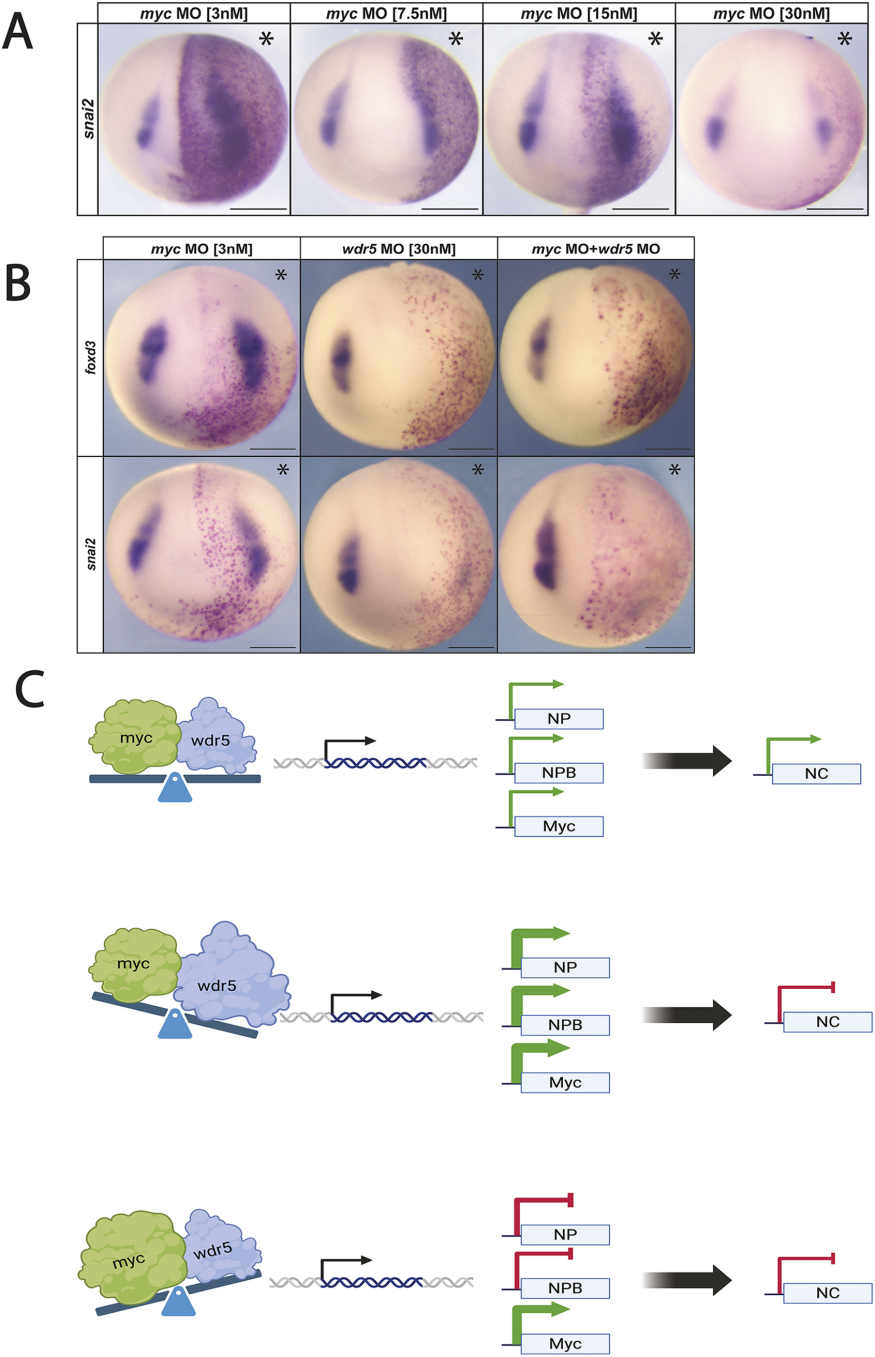
**Myc downregulation expands neural crest expression via a Wdr5-mediated mechanism.** (A) Lower concentrations of *myc* MO expand neural crest factor expression. (B) *wdr5* is required for *myc* MO-mediated expansion of neural crest gene expression. (C) Model depicting Wdr5- and Myc-mediated regulation of the neural crest: normal neural crest (NC) formation (top), loss of NC and prolonged neural plate (NP) and NPB (middle), loss of NC and prolonged Myc expression (bottom). Asterisks indicate the injected side of the embryo. Scale bars: 250 μm.

We interpret the ability of small increases of *wdr5* or the *wdr5-WBM^V268E^* mutant to expand the neural crest domain as increasing the number of cells with the relative levels of Myc and Wdr5 needed to promote neural crest gene expression. If this is the case, then decreasing the levels of Myc expression should have a similar effect. As 48 ng of *myc* MO led to a complete loss of neural crest gene expression, we examined the effects that progressively lower amounts of *myc* MO would have on neural crest formation. Reducing the amount of MO to 24 ng had little to no effect on neural crest (*snai2*: 84.4% unchanged, *n*=45). However, lower concentrations led to increased expression of *snai2*, consistent with our hypothesis (12 ng: 59% expansion, *n*=34; 6 ng: 79%, *n*=40; 2.4 ng: 86%, *n*=42) (Fig. 7A; Fig. S6A).

### Wdr5 is required for Myc-mediated promotion of neural crest formation

Given that decreasing *myc* expression levels promotes expression of neural crest factors, we next examined whether this ability of Myc to promote neural crest formation requires Wdr5. Embryos were injected with *myc* MO at a concentration that enhances neural crest formation (2.4 ng), *wdr5* MO at a concentration that inhibits neural crest formation (24 ng), or both MOs. When *wdr5* was depleted, reducing *myc* levels was no longer able to promote expression of the neural crest factors *foxd3* and *snai2*. (*myc* MO 2.4 ng+*wdr5* MO 24 ng: *foxd3*: 85%, *n*=17; *snai2*: 85%, *n*=13) (Fig. 7B; Fig. S6A). This evidence supports the hypothesis that Myc-mediated regulation of neural crest gene expression requires Wdr5.

## DISCUSSION

Wdr5 is a versatile scaffolding protein that functions in multiple cellular processes, including the maintenance of pluripotency in embryonic stem cells (Ang et al., 2011). Little is understood, however, about its functions in early embryonic development. Here we show that Wdr5 is essential for the formation of neural crest cells in both whole embryos and induced explants, and present evidence that Wdr5 plays roles in at least three distinct steps in the process by which initially pluripotent blastula stem cells transit to a neural crest stem cell state.

The most straight forward of these is in regulating the establishment of definitive neural crest cells. Here, Wdr5 functions with a known partner, Myc, to regulate expression of genes such as *snai2* and *foxd3* in a manner dependent on its WBM domain. Intriguingly, Wdr5 exhibited dose-dependent effects on neural crest gene expression. Lower doses enhanced neural crest formation, whereas higher doses inhibited it. Such biphasic effects have previously been reported for transcriptional regulators involved in pluripotency and differentiation, including Myc and Oct4 (Silva and Smith, 2008; Takahashi and Yamanaka, 2006). However, they are not characteristic of definitive neural crest factors such as Snai2 or Foxd3 (LaBonne and Bronner-Fraser, 2000; Heeg-Truesdell and LaBonne, 2004). Our findings suggest that Wdr5 levels must be precisely regulated to ensure proper stoichiometry with interacting partners such as Myc. Disruption of this stoichiometry can impede neural crest formation, reinforcing the idea that developmental decisions hinge critically on balanced protein interactions.

Our data show that Wdr5 and Myc can physically interact in early embryos, consistent with previous studies in cancer cells (Thomas et al., 2019). Similarly, our finding that co-expression of Myc and Wdr5 at specific doses rescued neural crest gene expression, whereas individually these factors suppressed neural crest formation, clearly highlights the functional synergy between these two proteins. Such synergy might be explained by Wdr5 enhancing the transcriptional regulatory functions of Myc, potentially by stabilizing complexes essential for neural crest-specific gene activation, as has been observed previously (Thomas et al., 2015b). Furthermore, the finding that depletion of Myc abolished the neural crest-promoting effects of Wdr5 further underscores the necessity of the Myc–Wdr5 complex for neural crest specification.

We found that both overexpression and depletion of Myc inhibited neural crest cell formation, whereas partial knockdown of Myc enhanced neural crest gene expression. Similar regulatory complexity has been observed in embryonic stem cells, where partial knockdown or controlled expression of Myc can promote differentiation into specific lineages (Cartwright et al., 2005). Thus, neural crest formation likely depends upon finely tuned levels of Myc activity, regulated by at least in part by Wdr5 interactions, to achieve proper developmental outcomes. Importantly, in human cancer cells, ChIP-Seq-based colocalization analyses found that approximately 80% of MYC-binding sites genome-wide are also occupied by WDR5 (Thomas et al., 2015b). Although disrupting the interaction between MYC and WDR5 has no effect on WDR5's own chromatin binding, it prevents MYC from binding the majority of its target sites. The requirement for precise stoichiometry between MYC and Wdr5 has been found in both cancer cells and embryonic stem cells (Guarnaccia et al., 2021; Thomas et al., 2015a).

Our mutational analyses also revealed a distinct role for the WIN domain of Wdr5, suggesting that both differential binding partners and regulatory outcomes are controlled by these domains. Mutation of the WIN domain inhibits the expression of *myc* itself whereas wild-type Wdr5 or the WBM mutant do not. These findings suggest that regulation of *myc* by Wdr5 is mediated by one or more proteins that bind the WIN domain. While it could be interactions with components of chromatin remodeling complexes, it could also be a transcription factor that can interact with the WIN domain. Intriguingly, Pax3 has several candidate WIN pocket-interacting domains, including an ARA motif at its C-terminus, and WIN-binding proteins frequently present short, exposed ARA/RxR motifs near accessible, disordered tails. Investigating a role for Wdr5-Pax3-mediated regulation of Myc will be an important future area of investigation. The results presented here show that Wdr5 can mediate distinct regulatory mechanisms via separate protein domains and suggest that compartmentalization of functions within Wdr5 could fine-tune transcriptional outcomes during early embryogenesis.

We also found that *wdr5* depletion led to expanded expression of NPB markers, suggesting that it plays a role in restricting the size of the border region endogenously. Surprisingly, however, increased *wdr5* expression also promoted NPB formation, as did both the WBM and WIN mutants. This provides evidence that there is a third mechanism by which Wdr5 regulates the process of neural crest formation that is independent of both the WBM and WIN domains. Indeed, roles for Wdr5 that do not require these domains have been previously described. For example, Wdr5 binds long noncoding RNAs via an RNA-binding pocket located between the 5th and 6th WD40 repeats, and this interaction has been shown to regulate the HoxA cluster (Lu et al., 2018; Wang et al., 2011). Furthermore, In *Drosophila*, WDR5 (WDS) binds NSL1 via a short linear motif on an outer blade surface distinct from the WIN or WBM sites (Dias et al., 2014). Future studies could profitably explore WBM/WIN-independent interacting factors responsible for NPB expansion. Disrupting such interaction could, for example, block cells from transiting to an epidermal state, thereby retaining them in an NPB state.

In addition to Myc, Wdr5 partners with at least one other transcription factor involved in controlling pluripotency. Wdr5 has been shown to bind Oct4 in embryonic stem cells, and is required for mediating self-renewal and reprogramming via the core embryonic stem cell transcriptional network (Ang et al., 2011). Although no evidence has been shown to implicate SoxB1 factors as Wdr5 binding partners, we can interpret the lack of effect on neural plate expression when *wdr5*-*WBM^V268E^* mRNA was overexpressed as an indication that interaction between Wdr5 and WBM-binding partners is required for effects on *sox3* expression. It is possible that a putative Wdr5 interaction with Pou5f3 could explain these findings given that Pou5f3 factors and Soxb1 factors are required for formation of the NPB (Schock et al., 2024). Given the persistent expression of both neural plate and NPB factors in Wdr5-depleted embryos, it is clear that Wdr5 is not required for formation of either of these cell types, and it will be important going forward to elucidate the mechanism by which Wdr5 restricts the boundaries of these domains endogenously,

Our findings are relevant to cancers such as neuroblastoma, melanoma and pheochromocytomas that arise from neural crest-derived cells (Greer et al., 1965; Guilmette and Sadow, 2019). Indeed, dysregulation of MYC is estimated to underlie about one-third of all cancer deaths (Tansey, 2014). The interaction between MYC and the WBM binding site of WDR5 has become a key drug target in treating MYC-related cancers (Thomas et al., 2019, 2015a,b). Likewise, the WIN binding site, which is the site that links WDR5 to chromatin and other partners, has been shown to affect a different subset of cancers when pharmacologically inhibited (Aho et al., 2019; Siladi et al., 2022). This is consistent with our findings that Wdr5 can regulate multiple aspects of developmental processes in a context-dependent manner. More generally, our findings that Wdr5 is essential for Myc-mediated regulation of neural crest development underscores the importance of precise stoichiometric ratios of transcriptional regulators to important developmental decisions in vertebrate embryos.

## MATERIALS AND METHODS

### Animals

All animal procedures were approved by the Institutional Animal Care and Use Committee, Northwestern University, and are in accordance with the National Institutes of Health's Guide for the Care and Use of Laboratory Animals.

### Embryological methods

Wild-type *Xenopus* laevis embryos were staged and collected in accordance with standard methods (Zahn et al., 2022) and cultured in 0.1× Marc's Modified Ringer's Solution (MMR) [0.1 M NaCl, 2 mM KCl, 1 mM MgSO_4_, 2 mM CaCl_2_, 5 mM HEPES (pH 7.8), 0.1 mM EDTA] until the desired stages. Embryos or blastula stem cell explants (also known as animal pole cell explants) used for WISH, HCR or immunofluorescence were fixed in 1× MEM [100 mM MOPS (pH 7.4), 2 mM EDTA, 1 mM MgSO_4_] with 3.7% formaldehyde and dehydrated in methanol prior to use. Embryos or blastula stem cell explants that underwent WISH were processed as described (LaBonne and Bronner-Fraser, 1998) and imaged using an Infinity 8-8 camera (Teledyne Lumenera). Results are representative of a minimum of three biological replicates.

Microinjection of mRNA or morpholinos was carried out at the 2- to 8-cell stage. mRNA was synthesized using an mMessage mMachine SP6 Transcription Kit (Invitrogen) and translation efficiency assessed by western blotting. Either β-galactosidase mRNA or fluorescein dextran were co-injected as a lineage tracer. For injection experiments, ∼417 pg of *myc* mRNA was injected. For *wdr5* (low) mRNA, 106 pg was injected versus ∼520 pg *wdr5* (high). For translation-blocking morpholinos (Gene Tools), the following amounts were injected per cell: *wdr5*: 24 ng; *myc*: 2.4 ng, 6 ng, 12 ng, 24 ng or 48 ng as noted in the text. Morpholino sequences were: *wdr5*, 5′GTTTCTTTTCTTCTGTTGCCATG-3′; *myc*, ′5-GGCGTTAAGAGGCATCTTTCC-3′.

### Blastula stem cell explant assays

Animal pole cells were manually dissected using forceps at blastula stage (stage 9). Manipulated embryos were injected into either both cells at the 2-cell stage or the animal cell at the 4- to 8-cell stage with either mRNA or morpholino. To induce an NPB/neural crest state, dissected explants were immediately cultured in 3 µM K02288 (Sigma-Aldrich) and 107 µM CHIR99021 (Sigma-Aldrich) in 1× MMR, as described by Huber and LaBonne (2024), and remained in pharmacological solution until the time of collection.

### Western blotting

Five whole embryos or ten explants were lysed in PBS+1% NP-40 supplemented with protease inhibitors [Complete Mini, EDTA-free tablet (Roche), leupeptin (Roche), aprotinin (Sigma-Aldrich) and phenylmethylsulfonylfluoride (PMSF; Sigma-Aldrich)]. SDS page and western blotting were used to detect proteins. The following primary antibodies were used: c-Myc 9E10 (1:3000; Santa Cruz Biotechnology; sc-40); FLAG M2 (1:3000; Sigma-Aldrich; F1804); actin (1:5000; Sigma-Aldrich; A2066). IRDyes (1:20,000; mouse-800 CW; rabbit-680 TL). The Odyssey platform (LI-COR Biosciences) were used to detect proteins and Image Studio Lite software was used to quantify protein. Results are representative of a minimum of three biological replicates.

### Co-IP

Five whole embryos were lysed in 1% NP-40 supplemented with protease inhibitors (see ‘Western blotting’ section). A 5% input was retained for western blot analysis and the remaining 95% incubated with c-Myc 9E10 antibody (1:500) for 1 h. Approximately 25-30 µl of PAS beads (Sigma-Aldrich; P3391) were added to the lysate and incubated for 2 h. Beads were washed with 1% NP-40 and remaining proteins eluted off the beads. Input and immunoprecipitation samples were analyzed by western blotting as described above. Results are representative of a minimum of three biological replicates.

### HCR

HCR methodologies were modified from those described by Choi et al. (2018). Whole embryos or explants were hybridized with DNA probe sets for *pax3*, *snai2* (Molecular Instruments) and incubated overnight at 37°C. Probe was removed, and samples were washed and then incubated overnight with DNA hairpins labeled with Alexa 647 or Alexa 546 (Molecular Instruments). Unbound hairpins were removed by four 15-min washes with 5× SSC and then samples were immediately mounted and imaged using a Nikon C2 upright confocal with two GaAsP detectors and four standard laser lines.

### Statistics

Quantification of western blotting and Co-IP experiments was performed from a minimum of three independent biological replicates, each representing protein lysates or pulldowns prepared from separate experimental collections. Band intensities were measured using Image Studio Lite software. For each replicate, the intensity of the target band was normalized to the loading control (actin) to account for gel-to-gel variation. For Co-IP experiments, normalized pulldown signal intensities were further divided by the corresponding input signal to correct for expression differences between conditions. Relative binding or expression levels were expressed as fold change relative to the wild-type *wdr5* (or control) condition, which was set to 1.0. Fold changes were log₂-transformed prior to statistical testing to stabilize variance and improve normality. Data were analyzed using unpaired, two-tailed Student's *t*-tests (for two-group comparisons) or one-way ANOVA followed by Tukey's multiple comparison test (for more than two groups). Tests were performed on log₂-transformed fold changes, and *P*-values <0.05 were considered statistically significant. In figures, data are presented as mean±s.e.m., and significance is denoted as *P<0.05, **P<0.01 or ***P<0.001.

## Supplementary Material

10.1242/develop.205204_sup1 Supplementary information

## References

[DEV205204C1] Aho, E. R., Wang, J., Gogliotti, R. D., Howard, G. C., Phan, J., Acharya, P., Macdonald, J. D., Cheng, K., Lorey, S. L., Lu, B. et al. (2019). Displacement of WDR5 from chromatin by a WIN site inhibitor with picomolar affinity. *Cell Rep.* 26, 2916-2928.e13. 10.1016/j.celrep.2019.02.047 30865883 PMC6448596

[DEV205204C2] Ang, Y. S., Tsai, S. Y., Lee, D. F., Monk, J., Su, J., Ratnakumar, K., Ding, J., Ge, Y., Darr, H., Chang, B. et al. (2011). Wdr5 mediates self-renewal and reprogramming via the embryonic stem cell core transcriptional network. *Cell* 145, 183-197. 10.1016/j.cell.2011.03.003 21477851 PMC3097468

[DEV205204C3] Bellmeyer, A., Krase, J., Lindgren, J. and LaBonne, C. (2003). The protooncogene c-Myc is an essential regulator of neural crest formation in *Xenopus*. *Dev. Cell* 4, 827-839. 10.1016/S1534-5807(03)00160-6 12791268

[DEV205204C4] Bernstein, B. E., Humphrey, E. L., Erlich, R. L., Schneider, R., Bouman, P., Liu, J. S., Kouzarides, T. and Schreiber, S. L. (2002). Methylation of histone H3 Lys 4 in coding regions of active genes. *Proc. Natl Acad. Sci. USA* 99, 8695-8700. 10.1073/pnas.082249499 12060701 PMC124361

[DEV205204C5] Bibonne, A., Néant, I., Batut, J., Leclerc, C., Moreau, M. and Gilbert, T. (2013). Three calcium-sensitive genes, fus, brd3 and wdr5, are highly expressed in neural and renal territories during amphibian development. *Biochim. Biophys. Acta* 1833, 1665-1671. 10.1016/j.bbamcr.2012.12.015 23287019

[DEV205204C6] Bogdanovic, O., Fernandez-Miñán, A., Tena, J. J., de la Calle-Mustienes, E., Hidalgo, C., van Kruysbergen, I., van Heeringen, S. J., Veenstra, G. J. and Gómez-Skarmeta, J. L. (2012). Dynamics of enhancer chromatin signatures mark the transition from pluripotency to cell specification during embryogenesis. *Genome Res.* 22, 2043-2053. 10.1101/gr.134833.111 22593555 PMC3460198

[DEV205204C7] Buitrago-Delgado, E., Nordin, K., Rao, A., Geary, L. and LaBonne, C. (2015). Shared regulatory programs suggest retention of blastula-stage potential in neural crest cells. *Science* 348, 1332-1335. 10.1126/science.aaa3655 25931449 PMC4652794

[DEV205204C8] Buitrago-Delgado, E., Schock, E. N., Nordin, K. and LaBonne, C. (2018). A transition from SoxB1 to SoxE transcription factors is essential for progression from pluripotent blastula cells to neural crest cells. *Dev. Biol.* 444, 50-61. 30144418 10.1016/j.ydbio.2018.08.008 PMC8022798

[DEV205204C9] Cartwright, P., McLean, C., Sheppard, A., Rivett, D., Jones, K. and Dalton, S. (2005). LIF/STAT3 controls ES cell self-renewal and pluripotency by a Myc-dependent mechanism. *Development* 132, 885-896. 10.1242/dev.01670 15673569

[DEV205204C48] Choi, H. M. T., Schwarzkopf, M., Fornace, M. E., Acharya, A., Artavanis, G., Stegmaier, J., Cunha, A. and Pierce, N. A. (2018). Third-generation in situ hybridization chain reaction: multiplexed, quantitative, sensitive, versatile, robust. *Development* 145, dev165753. 10.1242/dev.165753 29945988 PMC6031405

[DEV205204C10] Dharmarajan, V., Lee, J. H., Patel, A., Skalnik, D. G. and Cosgrove, M. S. (2012). Structural basis for WDR5 interaction (Win) motif recognition in human SET1 family histone methyltransferases. *J. Biol. Chem.* 287, 27275-27289. 10.1074/jbc.M112.364125 22665483 PMC3431640

[DEV205204C11] Dias, J., Van Nguyen, N., Georgiev, P., Gaub, A., Brettschneider, J., Cusack, S., Kadlec, J. and Akhtar, A. (2014). Structural analysis of the KANSL1/WDR5/KANSL2 complex reveals that WDR5 is required for efficient assembly and chromatin targeting of the NSL complex. *Genes Dev.* 28, 929-942. 10.1101/gad.240200.114 24788516 PMC4018492

[DEV205204C12] Dou, Y., Milne, T. A., Ruthenburg, A. J., Lee, S., Lee, J. W., Verdine, G. L., Allis, C. D. and Roeder, R. G. (2006). Regulation of MLL1 H3K4 methyltransferase activity by its core components. *Nat. Struct. Mol. Biol.* 13, 713-719. 10.1038/nsmb1128 16878130

[DEV205204C13] Dovey, O. M., Foster, C. T. and Cowley, S. M. (2010). Histone deacetylase 1 (HDAC1), but not HDAC2, controls embryonic stem cell differentiation. *Proc. Natl. Acad. Sci. USA* 107, 8242-8247. 10.1073/pnas.1000478107 20404188 PMC2889513

[DEV205204C14] Geary, L. and LaBonne, C. (2018). FGF mediated MAPK and PI3K/Akt Signals make distinct contributions to pluripotency and the establishment of Neural Crest. *eLife* 7, e33845. 10.7554/eLife.33845 29350613 PMC5790379

[DEV205204C15] Greer, M., Anton, A. H., Williams, C. M. and Echevarria, R. A. (1965). Tumors of neural crest origin: biochemical and pathological correlation. *Arch. Neurol.* 13, 139-148. 10.1001/archneur.1965.00470020029004 14318483

[DEV205204C49] Groves, A. K. and LaBonne, C. (2014). Setting appropriate boundaries: fate, patterning and competence at the neural plate border. *Dev. Biol.* 389, 2-12. 10.1016/j.ydbio.2013.11.027 24321819 PMC3972267

[DEV205204C16] Guarnaccia, A. D. and Tansey, W. P. (2018). Moonlighting with WDR5: a cellular multitasker. *J. Clin. Med.* 7, 21. 10.3390/jcm7020021 29385767 PMC5852437

[DEV205204C17] Guarnaccia, A. D., Rose, K. L., Wang, J., Zhao, B., Popay, T. M., Wang, C. E., Guerrazzi, K., Hill, S., Woodley, C. M., Hansen, T. J. et al. (2021). Impact of WIN site inhibitor on the WDR5 interactome. *Cell Rep.* 34, 108636. 10.1016/j.celrep.2020.108636 33472061 PMC7871196

[DEV205204C18] Guilmette, J. and Sadow, P. M. (2019). A guide to pheochromocytomas and paragangliomas. *Endocr Pathol.* 12, 951-965. 10.1016/j.path.2019.08.009 PMC7403630 31672301

[DEV205204C46] Heeg-Truesdell, E. and LaBonne, C. (2004). A slug, a fox, a pair of sox: transcriptional responses to neural crest inducing signals. *Birth Defects Res. C Embryo Today* 72, 124-139. 10.1002/bdrc.20011 15269887

[DEV205204C19] Huber, P. B. and LaBonne, C. (2024). Small molecule-mediated reprogramming of *Xenopus* blastula stem cells to a neural crest state. *Dev. Biol.* 505, 34-41. 10.1016/j.ydbio.2023.10.004 37890713 PMC11541498

[DEV205204C20] Huber, P. B., Rao, A. and LaBonne, C. (2024). BET activity plays an essential role in control of stem cell attributes in *Xenopus*. *Development* 151, dev202990. 10.1242/dev.202990 38884356 PMC11266789

[DEV205204C21] Johnson, K., Freedman, S., Braun, R. and LaBonne, C. (2022). Quantitative analysis of transcriptome dynamics provides novel insights into developmental state transitions. *BMC Genomics* 23, 723. 10.1186/s12864-022-08992-8 36273135 PMC9588240

[DEV205204C47] LaBonne, C. and Bronner-Fraser, M. (1998). Neural crest induction in Xenopus: evidence for a two-signal model. *Development* 125, 2403-2414. 10.1242/dev.125.13.2403 9609823

[DEV205204C50] LaBonne, C. and Bronner-Fraser, M. (2000). Snail-related transcriptional repressors are required in Xenopus for both the induction of the neural crest and its subsequent migration. *Dev. Biol.* 221, 195-205. 10.1006/dbio.2000.9609 10772801

[DEV205204C22] Le Douarin, N. and Kalcheim, C. (1999). *The Neural Crest*, 2nd edn. Cambridge University Press.

[DEV205204C51] Light, W., Vernon, A. E., Lasorella, A., Iavarone, A. and LaBonne, C. (2005). Xenopus Id3 is required downstream of Myc for the formation of multipotent neural crest progenitor cells. *Development* 132, 1831-1841. 10.1242/dev.01734 15772131

[DEV205204C23] Lu, K., Tao, H., Si, X. and Chen, Q. (2018). The histone H3 lysine 4 presenter WDR5 as an oncogenic protein and novel epigenetic target in cancer. *Front. Oncol.* 8, 502. 10.3389/fonc.2018.00502 30488017 PMC6246693

[DEV205204C24] Patel, A., Dharmarajan, V. and Cosgrove, M. S. (2008a). Structure of WDR5 bound to mixed lineage leukemia protein-1 peptide. *J. Biol. Chem.* 283, 32158-32161. 10.1074/jbc.C800164200 18829459

[DEV205204C25] Patel, A., Vought, V. E., Dharmarajan, V. and Cosgrove, M. S. (2008b). A conserved arginine-containing motif crucial for the assembly and enzymatic activity of the mixed lineage leukemia protein-1 core complex. *J. Biol. Chem.* 283, 32162-32175. 10.1074/jbc.M806317200 18829457

[DEV205204C26] Prasad, M. S., Sauka-Spengler, T. and LaBonne, C. (2012). Induction of the neural crest state: control of stem cell attributes by gene regulatory, post-transcriptional and epigenetic interactions. *Dev. Biol.* 366, 10-21. 10.1016/j.ydbio.2012.03.014 22583479 PMC3354335

[DEV205204C27] Rao, A. and LaBonne, C. (2018). Histone deacetylase activity has an essential role in establishing and maintaining the vertebrate neural crest. *Development* 145, dev163386. 10.1242/dev.163386 30002130 PMC6110147

[DEV205204C28] Rigney, S., York, J. R. and LaBonne, C. (2025). Krüppel-like factors play essential roles in regulating pluripotency and the formation of neural crest stem cells. *Development* 152, dev204634. 10.1242/dev.204634 40292574 PMC12070069

[DEV205204C29] Ruthenburg, A. J., Wang, W., Graybosch, D. M., Li, H., Allis, C. D., Patel, D. J. and Verdine, G. L. (2006). Histone H3 recognition and presentation by the WDR5 module of the MLL1 complex. *Nat. Struct. Mol. Biol.* 13, 704-712. 10.1038/nsmb1119 16829959 PMC4698793

[DEV205204C30] Schock, E. N., York, J. R. and LaBonne, C. (2023). The developmental and evolutionary origins of cellular pluripotency in the vertebrate neural crest. *Semin. Cell Dev. Biol.* 138, 36-44. 10.1016/j.semcdb.2022.04.008 35534333 PMC11513157

[DEV205204C31] Schock, E. N., York, J. R., Li, A. P., Tu, A. Y. and LaBonne, C. (2024). SoxB1 transcription factors are essential for initiating and maintaining neural plate border gene expression. *Development* 151, dev202693. 10.1242/dev.202693 38940470 PMC11369808

[DEV205204C32] Schuetz, A., Allali-Hassani, A., Martín, F., Loppnau, P., Vedadi, M., Bochkarev, A., Plotnikov, A. N., Arrowsmith, C. H. and Min, J. (2006). Structural basis for molecular recognition and presentation of histone H3 by WDR5. *EMBO J.* 25, 4245-4252. 10.1038/sj.emboj.7601316 16946699 PMC1570438

[DEV205204C33] Siladi, A. J., Wang, J., Florian, A. C., Thomas, L. R., Creighton, J. H., Matlock, B. K., Flaherty, D. K., Lorey, S. L., Howard, G. C., Fesik, S. W. et al. (2022). WIN site inhibition disrupts a subset of WDR5 function. *Sci. Rep.* 12, 1848. 10.1038/s41598-022-05947-9 35115608 PMC8813994

[DEV205204C34] Silva, J. and Smith, A. (2008). Capturing pluripotency. *Cell* 132, 532-536. 10.1016/j.cell.2008.02.006 18295569 PMC2427053

[DEV205204C35] Takahashi, K. and Yamanaka, S. (2006). Induction of pluripotent stem cells from mouse embryonic and adult fibroblast cultures by defined factors. *Cell* 126, 663-676. 10.1016/j.cell.2006.07.024 16904174

[DEV205204C36] Tansey, W. P. (2014). Mammalian MYC proteins and cancer. *N. J. Sci.* 2014, 757534. 10.1155/2014/757534

[DEV205204C37] Thomas, L. R., Foshage, A. M., Weissmiller, A. M. and Tansey, W. P. (2015a). The MYC-WDR5 nexus and cancer. *Cancer Res.* 75, 4012-4015. 10.1158/0008-5472.Can-15-1216 26383167 PMC4592407

[DEV205204C38] Thomas, L. R., Wang, Q., Grieb, B. C., Phan, J., Foshage, A. M., Sun, Q., Olejniczak, E. T., Clark, T., Dey, S., Lorey, S. et al. (2015b). Interaction with WDR5 promotes target gene recognition and tumorigenesis by MYC. *Mol. Cell* 58, 440-452. 10.1016/j.molcel.2015.02.028 25818646 PMC4427524

[DEV205204C39] Thomas, L. R., Adams, C. M., Wang, J., Weissmiller, A. M., Creighton, J., Lorey, S. L., Liu, Q., Fesik, S. W., Eischen, C. M. and Tansey, W. P. (2019). Interaction of the oncoprotein transcription factor MYC with its chromatin cofactor WDR5 is essential for tumor maintenance. *Proc. Natl. Acad. Sci. USA* 116, 25260-25268. 10.1073/pnas.1910391116 31767764 PMC6911241

[DEV205204C40] Wang, K. C., Yang, Y. W., Liu, B., Sanyal, A., Corces-Zimmerman, R., Chen, Y., Lajoie, B. R., Protacio, A., Flynn, R. A., Gupta, R. A. et al. (2011). A long noncoding RNA maintains active chromatin to coordinate homeotic gene expression. *Nature* 472, 120-124. 10.1038/nature09819 21423168 PMC3670758

[DEV205204C41] Watanabe, A., Yamada, Y. and Yamanaka, S. (2013). Epigenetic regulation in pluripotent stem cells: a key to breaking the epigenetic barrier. *Philos. Trans. R. Soc. Lond. B Biol. Sci.* 368, 20120292. 10.1098/rstb.2012.0292 23166402 PMC3539367

[DEV205204C42] Wysocka, J., Swigut, T., Milne, T. A., Dou, Y., Zhang, X., Burlingame, A. L., Roeder, R. G., Brivanlou, A. H. and Allis, C. D. (2005). WDR5 associates with histone H3 methylated at K4 and is essential for H3 K4 methylation and vertebrate development. *Cell* 121, 859-872. 10.1016/j.cell.2005.03.036 15960974

[DEV205204C43] York, J. R., Rao, A., Huber, P. B., Schock, E. N., Montequin, A., Rigney, S. and LaBonne, C. (2024). Shared features of blastula and neural crest stem cells evolved at the base of vertebrates. *Nat. Ecol. Evol.* 8, 1680-1692. 10.1038/s41559-024-02476-8 39060477 PMC11520720

[DEV205204C44] Zahn, N., James-Zorn, C., Ponferrada, V. G., Adams, D. S., Grzymkowski, J., Buchholz, D. R., Nascone-Yoder, N. M., Horb, M., Moody, S. A., Vize, P. D. et al. (2022). Normal table of *Xenopus* development: a new graphical resource. *Development* 149, dev200356. 10.1242/dev.200356 35833709 PMC9445888

[DEV205204C45] Zhang, P., Lee, H., Brunzelle, J. S. and Couture, J. F. (2012). The plasticity of WDR5 peptide-binding cleft enables the binding of the SET1 family of histone methyltransferases. *Nucleic Acids Res.* 40, 4237-4246. 10.1093/nar/gkr1235 22266653 PMC3351189

