# Supplementary material for: Wdr5 and Myc cooperate to regulate formation of neural crest stem cells
Source: Development. 2026 Jan 23;153(2):dev205204. doi: 10.1242/dev.205204 (PMC12863299; doi:10.1242/dev.205204)
Supplement: Supplementary information [file develop-153-205204-s1.pdf]

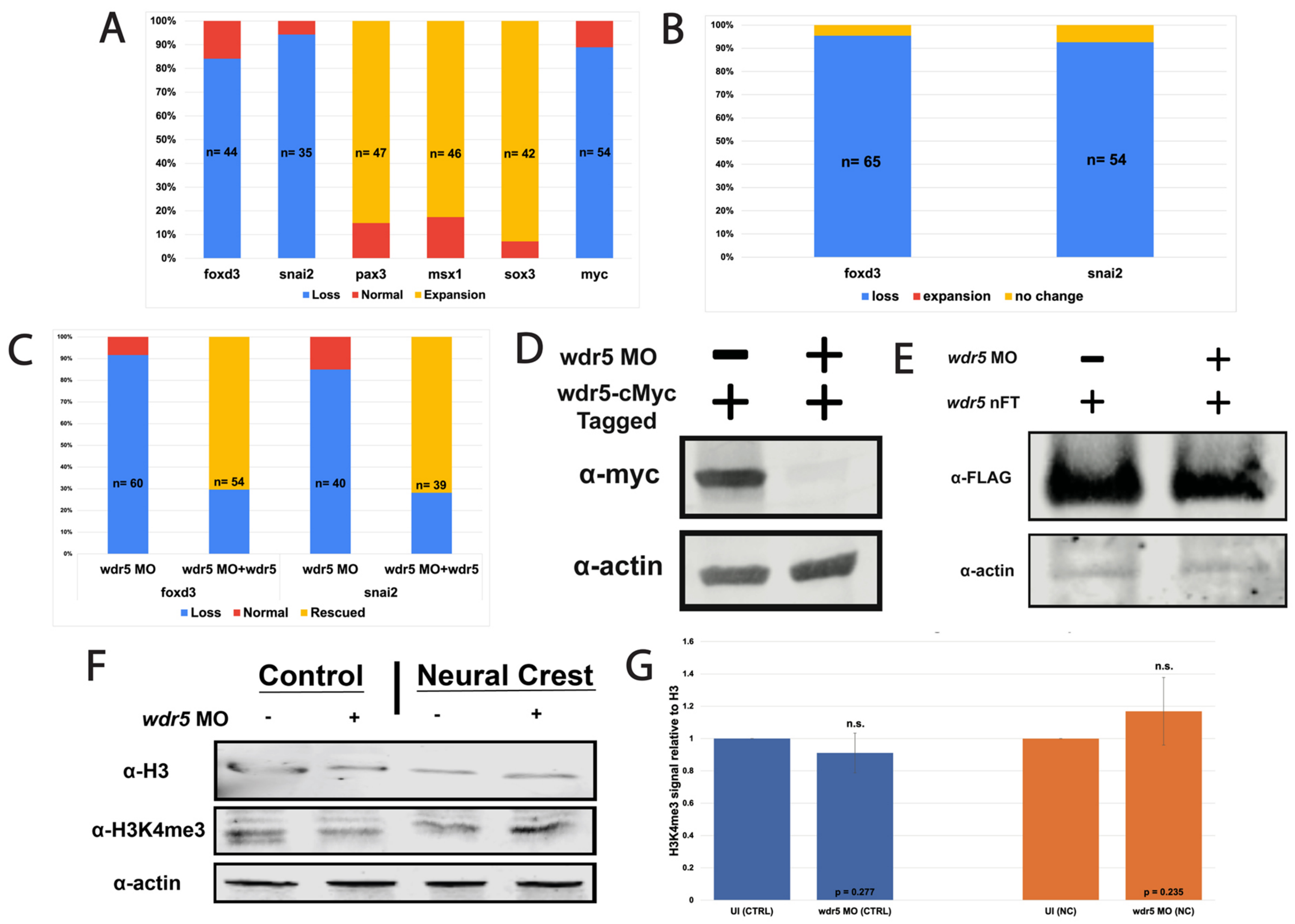

Fig. S1. (A) Percentage bar graph representing whole embryo phenotype counts for wdr5 MO injected embryos. (B) Percentage bar graph representing phenotype counts for wdr5 MO in-jected neural crest explants. (C) Percentage bar graph representing phenotype counts for wdr5 MO and wdr5 MO + wdr5 mRNAinjected embryos. (D) Western blot depicting specificity of wdr5 morpholi-no. (E) Western blot depicting expression of n-terminally FLAG-tagged wdr5 persists in the absence or presence of wdr5 morpholino. (F) Western blot of global levels of H3K4me3 compared to total H3 in control vs neural crest caps, with and without wdr5 morpholino. (G) Quantification of global levels of H3K4me3 compared to total H3 in control vs neural crest caps, with and without wdr5 morpholino. (Av-erage of 3 replicates. n.s., p>0.05)

Development • Supplementary information

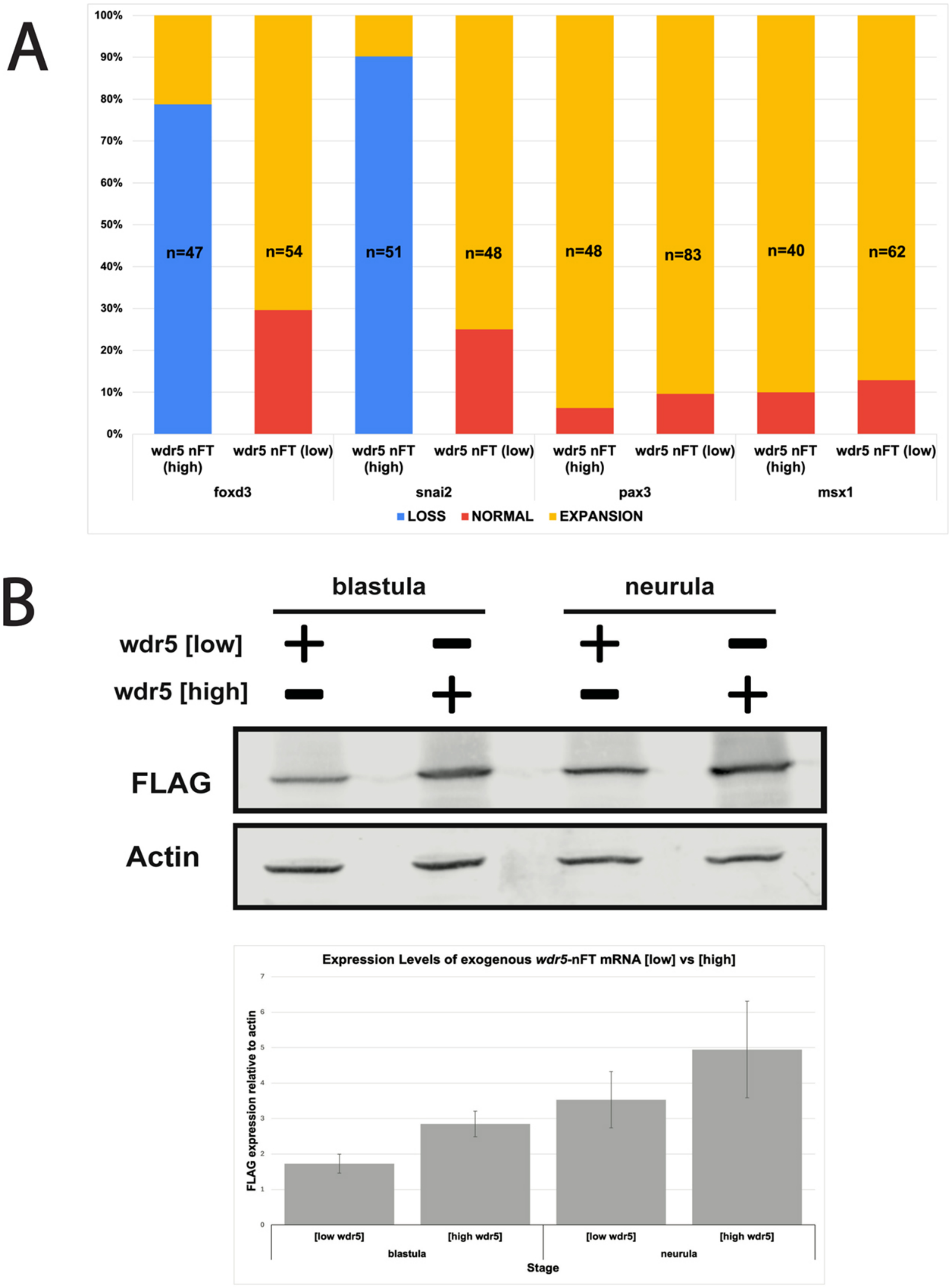

Fig. S2. (A) Percentage bar graph representing whole embryo phenotype counts for wdr5 nFT injected embryos (low vs high dose). (B) Western blot and quantification of difference in low vs high concentrations of wdr5-nFT mRNA.

Development • Supplementary information

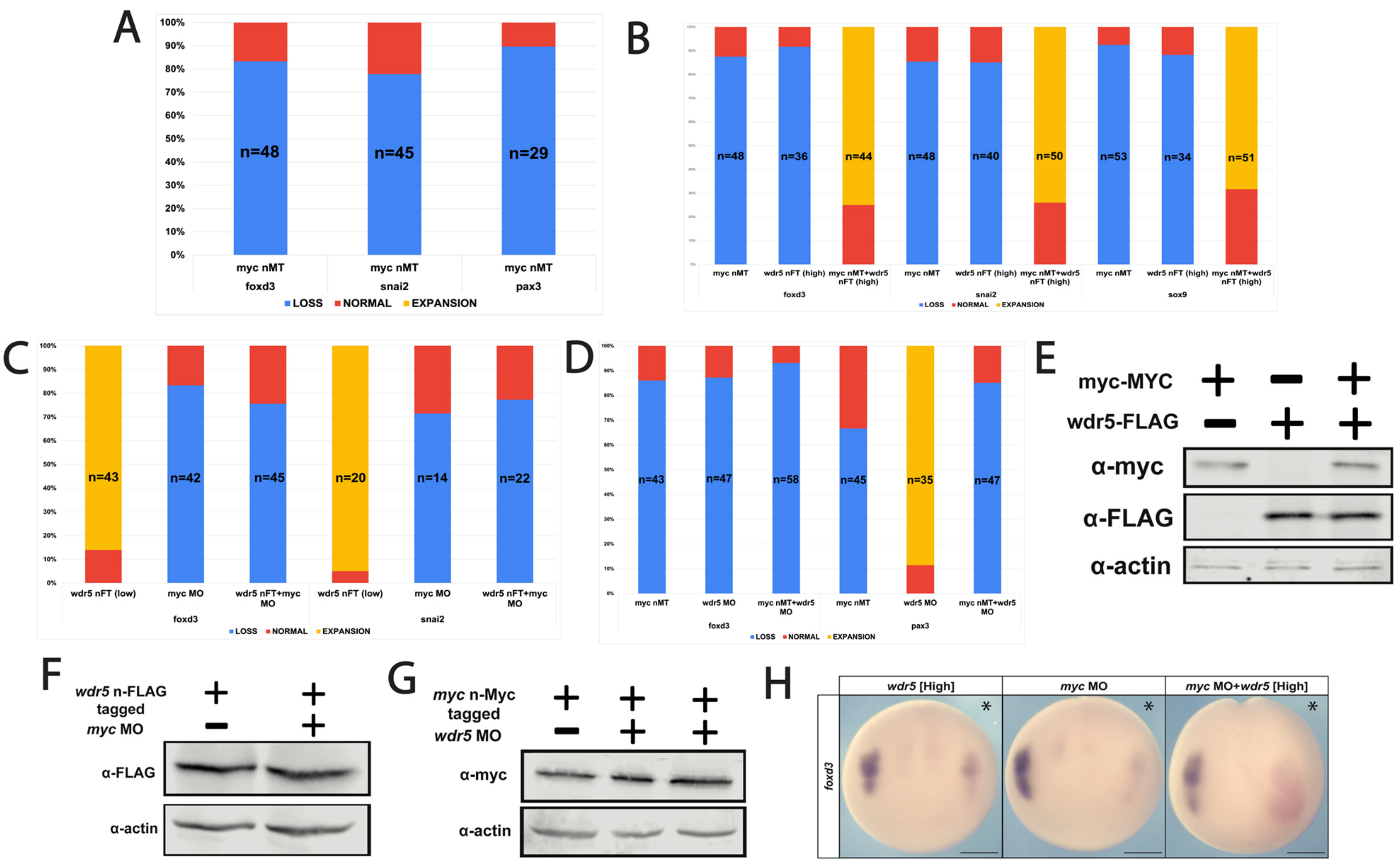

Fig. S3. (A) Percentage bar graph representing whole embryo phenotype counts for myc-nMT injected embryos. (B) Percentage bar graph representing whole embryo phenotype counts for myc nMT, wdr5 nFT, and myc+wdr5 injected embryos. (C) Percentage bar graph representing whole embryo phenotype counts for wdr5 nFT, myc MO, and wdr5 nFT+myc MO injected embryos. (D) Percentage bar graph representing whole embryo phenotype counts for myc nMT, wdr5 MO, and myc nMT+wdr5 MO injected embryos. (E) Western blot depitcing equivalent expression of wdr5-FLAG and myc-Myc in single and co-injection conditions. (F) Western blot depicting persistence of wdr5-FLAG expression in the presence and absence of myc morpholino. (G) Western blot depicting persistence of myc-MYC expression in the presence and absence of wdr5 morpholino. (H) High levels of wdr5 do not require myc expression to inhibit neural crest gene expression.

Development • Supplementary information

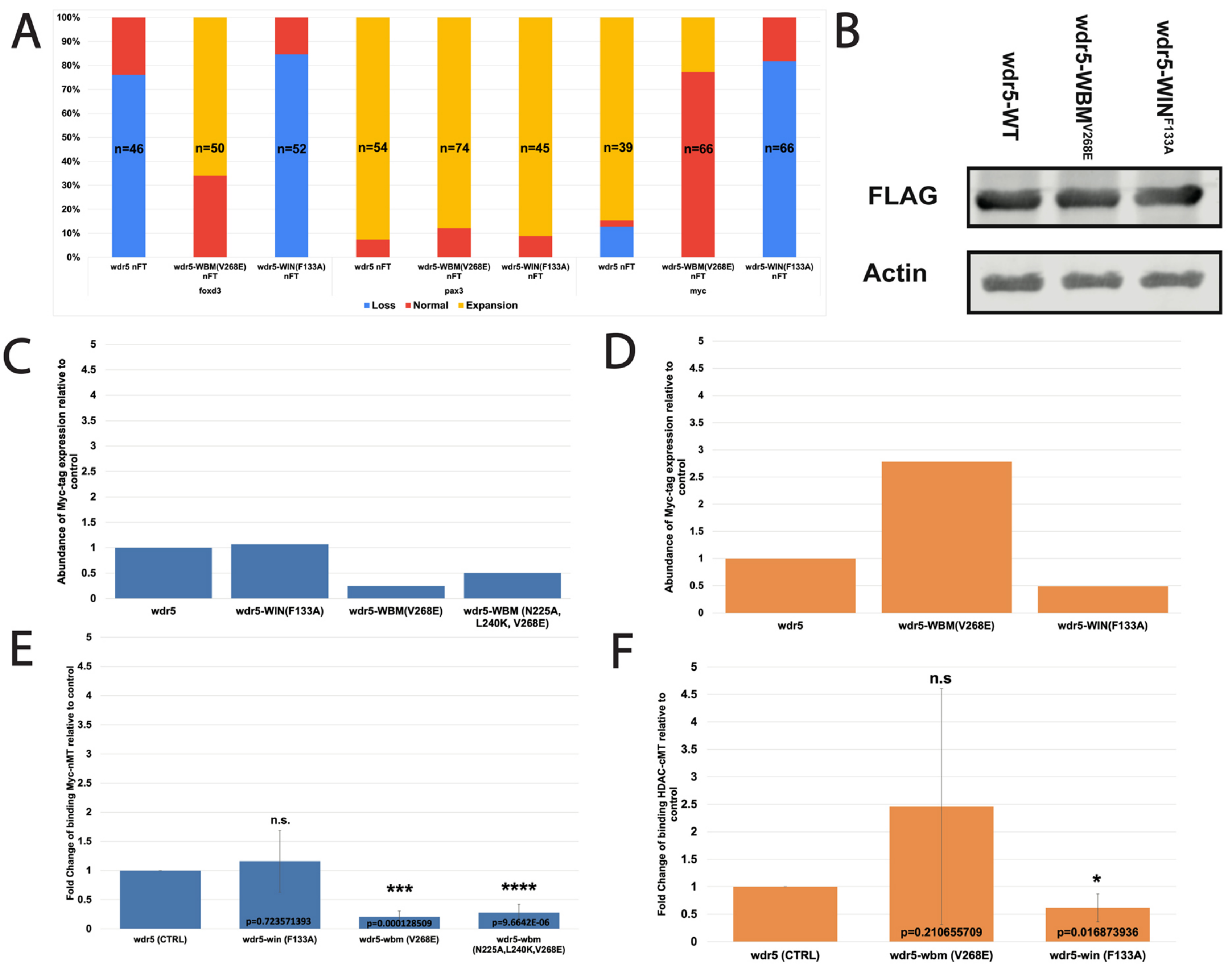

Fig. S4. (A) Percentage bar graph representing whole embryo phenotype counts for wdr5, wdr5-WBM^{V268}, and wdr5-WIN^{F133A} injected embryos. (B) Western blot showing equivalent expres-sion of wdr5-WT, wdr5- WBM^{V268E}, wdr5-WIN^{F133A} FLAG tag. (C) Co-IP binding analysis of myc+wdr5, wdr5-WBM^{V268}, wdr5-WBM^{N225A,L240K,V268E}, or wdr5-WIN^{F133A} pulldown abundance. (D) Co-IP binding analy-sis of myc+wdr5, wdr5-WBM^{V268}, wdr5-WBM^{N225A,L240K,V268E}, or wdr5-WIN^{F133A} pulldown abundance. (E) Quantification of Co-IP binding analysis of myc+wdr5, wdr5-WBM^{V268}, wdr5-WBM^{N225A,L240K,V268E}, or wdr5-WIN^{F133A} pulldown abundance(Average of 3 replicates). (F) Quantification of Co-IP binding analysis of hdac1+wdr5, wdr5-WBM^{V268}, or wdr5-WIN^{F133A} pulldown abundance (Average of 3 replicates).

Development • Supplementary information

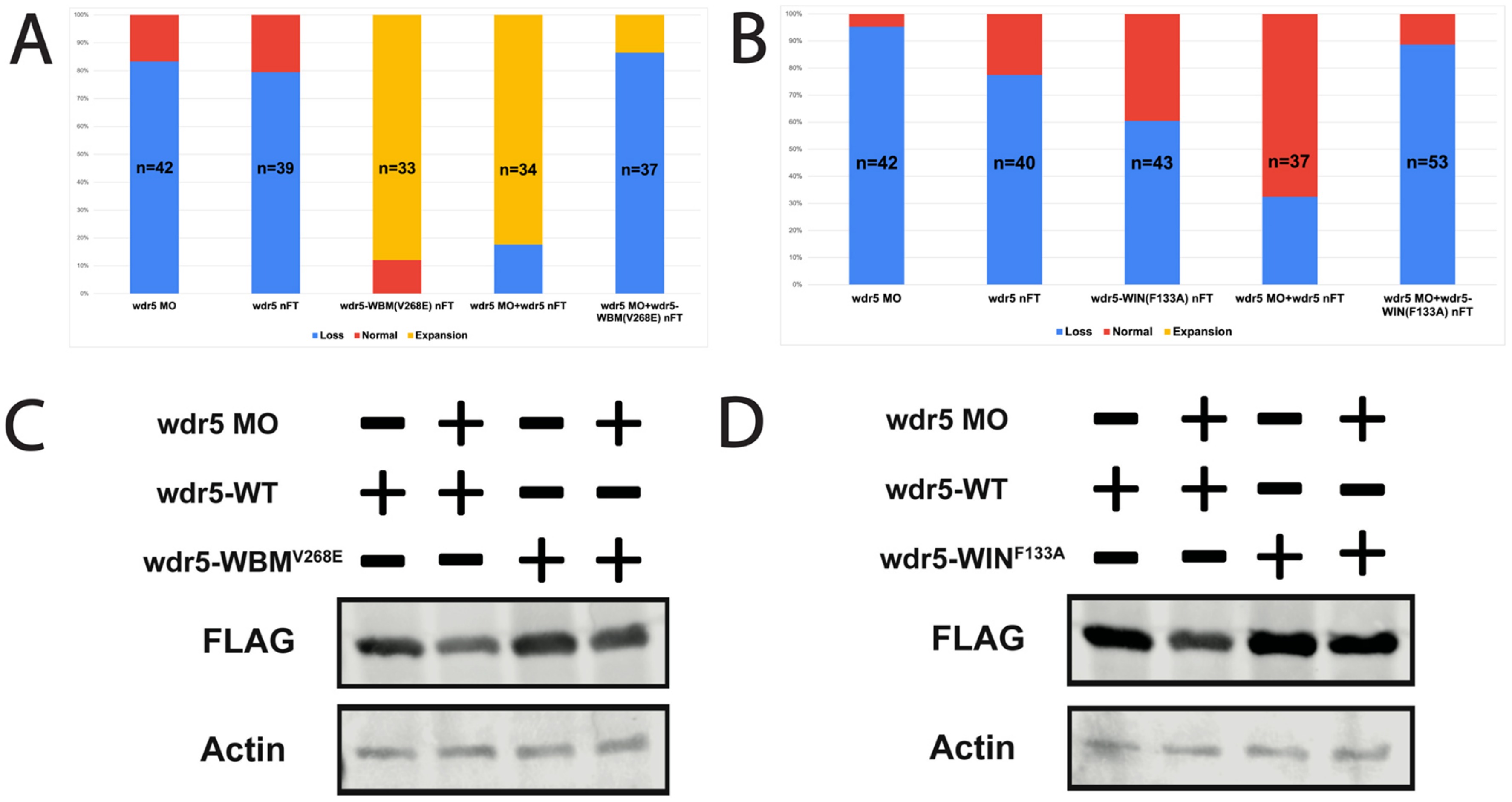

Fig. S5. (A) Percentage bar graph representing whole embryo phenotype counts for wdr5 MO, wdr5 nFT, wdr5-WBM^{V268E} nFT,wdr5 MO+wdr5 nFT, and wdr5 MO+wdr5-WBM^{V268E} injected embryos. (B) Percentage bar graph representing whole embryo phenotype counts for wdr5 MO, wdr5 nFT, wdr5-WIN^{F133A} nFT,wdr5 MO+wdr5 nFT, and wdr5 MO+wdr5-WIN^{F133A} inject-ed embryos. (C) Western blot showing equivalent expression of wdr5-WT and wdr5-WBM^{V268E} in the absence of endogenous wdr5 expression. (D) Western blot showing equivalent expression of wdr5-WT and wdr5-WIN^{F133A} in the absence of endogenous wdr5 expression.

Development • Supplementary information

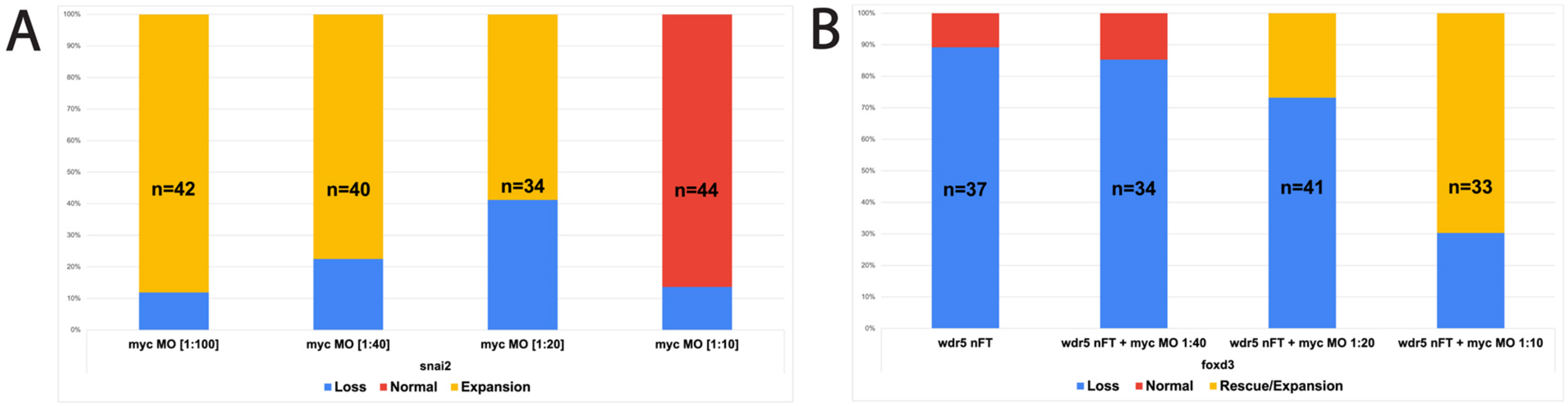

Fig. S6. (A) Percentage bar graph representing whole embryo phenotype counts for myc MO injected embryos at varying concentrations. (B) Percentage bar graph representing whole embryo phenotype counts for embryos injected with either wdr5 nFT, wdr5 nFT+myc MO at varying concentrations.

Development • Supplementary information
